# Comparative Analysis and Systematic Mapping of the Labial Sensilla in the Nepomorpha (Heteroptera: Insecta)

**DOI:** 10.1155/2013/518034

**Published:** 2013-07-01

**Authors:** Jolanta Brożek

**Affiliations:** Department of Zoology, Faculty of Biology and Environmental Protection, University of Silesia, Bankowa Street 9, 40-007 Katowice, Poland

## Abstract

The present study provides new data concerning the morphology and distribution of the labial sensilla of 55 species of 12 nepomorphan families (Heteroptera: Nepomorpha) using the scanning electron microscope. On the labial tip, three morphologically distinct types of chemosensilla have been identified: two types of papillae sensilla and one type of peg-in-pit sensilla. Twenty-one morphologically distinct types of the mechanosensilla as well as two types of the trichoid sensilla (contact-chemoreceptive sensillum) have been identified on all labial segments in representatives of subfamilies. In Nepomorpha, morphological ground plan of the labial sensory structures is represented by an apical sensory field with 10–13 pairs of papillae sensilla and the peg-in-pit ones placed more laterally; numerous trichoid sensilla are placed on the IV segment; the chaetica sensilla are present and placed in groups or rows distributed along the labium near the labial groove on the dorsal side, and also several chaetica sensilla are unevenly scattered on the surface of that segment; the cupola and peg sensilla are numerous and evenly scattered on the fourth labial segment; the prioprerecptive sensilla, one pair is positioned on the dorsal side and on the fourth segment of the labium. The new apomorphical characters have been established for the labial sensilla in the Nepomorpha.

## 1. Introduction

The mouthparts of the hemipteran insects are organs connected with feeding, comprising the unpaired labrum in the front, a median hypopharynx behind the mouth, a pair of mandibles and maxillae laterally, and usually a segmented labium [1, 2]. The sensory system, which mediates feeding, is used for external detection of the surface of plants or animals and host acceptance by antennal or labial sensilla (gustative and contact-chemoreceptive ones). All hemipterans have a large number of various antennal sensilla, and they are probably used to detect olfactory cues during long-distance orientation to the host plants. In the case of the labium of the hemipteran insects, the apical segment with the apical sensilla plays a role in recognizing the outer surface of the plant or animal food source, while the inner sensory organ (e.g., precibarial sensilla) comes directly into contact with the food as it is ingested [3–5]. These sensilla belong to the group of chemosensilla (gustative sensilla); they have a single relatively large pore at the tip (i.e., uniporous sensilla) and are sunken in inflexible sockets, distinguishing them from olfactory sensilla that have many small pores; they are called multiporous sensilla and are also sunken in inflexible sockets [6, 7]. Contact chemoreceptives have various forms, which are described as the trichoids, bristles, pegs, basiconica, spots, pits, and domes. All of these have a pore on the exterior and are innervated by neurons with features characteristic of chemo- and mechanoreceptors; their base can be distinctly sunken in flexible sockets [4, 6, 8]. Moreover, the surfaces of all labial segments are usually equipped with various sensory structures by which different signals from the environment are detected. The most common among those is the tactile structures which greatly vary in form, including the hair-shaped bristles, chaetica, trichodea, trichobothria, pegs, dome-like, and campaniform sensilla and representing a large group of mechanosensilla. These sensilla are without pores and are attached to the labium in a socket by an articulation membrane [4, 6, 8].

In most of the Nepomorpha, the labium consists of four segments more or less extended and is directly exposed to water [1, 2, 9]. Solely in the Corixoidea, the labium is more or less triangular, much shorter and dorsally bears a deep stylet groove flanked by a series of sclerotized transverse bands [10–12]. These transverse bars are separated from each other by slightly sclerotized membranes in which the sense organs are located [10, 13, 14]. In particular, in the heteropterans the tips of the labium (two lateral lobes) have gustative sensilla, frequently the contact chemoreceptive sensilla, and rarely also the olfactive, hygro-, and thermoreceptive sensilla as well as the tactile sensilla [1, 15, 16]. Several types of these sensilla on the labial tip have been described in many species of the Pentatomomorpha [17–23], the Nepomorpha [1, 24] and the Gerromorpha [Brożek and Zettel, in prep]. A full documentation of different cuticular hairy structures of the mechanosensilla and chemosensilla on all segments of the labium in several species of the Triatominae (Cimicomorpha: Reduviidae) has been presented by Catalá [25] and on distal segment of the labium in the Peiratinae (Cimicomorpha: Reduviidae) by Brożek and Chłond [15]. A comprehensive study of the Triatominae and the Peiratinae species has shown that the types of the labial mechanosensilla and their numbers are similar in individual species. Such information can be used to compare their labial sensilla with those found in other groups of the Heteroptera. Documentation of the presence of different types of labial sensilla in a large set of taxa will result in a concise picture of the systematic distribution of this structure across the Nepomorpha, which may prove useful for future systematic analysis. The scanning electron microscopy will refine the picture of fine structural details of the labial sensilla in the studied nepomorphan groups. 

The study aims to provide an insight into the sensilla on the labium in representatives of the Nepomorpha and attempts to clarify if the discovered types of the sensilla and their distribution have a phylogenetic value for the water bugs.

## 2. Materials and Methods

### 2.1. Taxon Samples

This study of labial sensilla was based on dry material consisting of adult specimens from the collections of the Natural History Museum in Vienna, Zoological Museum of the State Moscow University, and the Paleontological Institute of the Russian Academy of Sciences in Moscow. The basal part of the head with a part of the rostrum or the whole specimens was glued onto a scanning electron microscope stub. The labial sensilla used for SEM photographs were not coated; the photographs were taken with a Hitachi scanning electron microscope, with the samples placed in the low pressure chamber.

Classification and order of families and subfamilies listed below are the same as that established by Štys and Jansson [26], except for the Micronectidae and the Diaprepocoridae which have been elevated to the rank of family [27].

In the part of discussion, the ground plan of the morphological labial sensilla structures and the preliminary estimation of the characters of the labial sensilla in respect to phylogenetic value are compared with the basal model within the group (i.e., the basal taxa of the Nepidae and Belostomatidae) to the more diverse forms of these structures in more evolutionarily advanced groups (i.e., Corixidae, Ochteridae, Gelastocoridae, Aphelocheiridae, Naucoridae, Pleidae, Helotrephidae, and Notonectidae, resp.).

Figures 1–8 are placed in the results, and Figures 9–31 are placed in the appendix.

A list of taxa whose sensilla have been studied is given below.

*Nepidae. *Nepinae: *Curicta granulosa* De Carlo, *Borborophyes mayri* Stål, *Laccotrephes japonensis* Scott, *Nepa cinerea* Linnaeus; Ranatrinae: *Cercotmetus asiaticus* Amyot & Serville, *Ranatra chinensis* Mayr.

*Belostomatidae.* Belostomatinae: *Belostoma flumineum* Say, *Dienostoma dilatatum* (Say), *Appasus major *Esaki, *Hydrocyrius colombiae* Spinola, *Limnogeton fieberi* Mayr, Lethocerinae:* Lethocerus deyrollei *(Vuillefroy).

*Ochteridae. Ochterus marginatus *(Latreille), *Ochterus piliferus *Kormilev.

*Gelastocoridae. Gelastocoris oculatus *(Fabricius), *Nerthra nepaeformis *(Fabricius), *Nerthra macrothorax* (Montrouzier).

*Aphelocheiridae. Aphelocheirus variegatus* Kiritschenko, *Aphelocheirus aestivalis *(Fabricius).

*Naucoridae.* Cheirochelinae: *Cheirochela feana* Montandon*, Gestroiella limnocoroides *Montandon, *Coptocatus oblongulus *Montandon*, Coptocatus kinabalu *Polhemus,* Tanycricos longiceps *La Rivers, Laccocorinae*: Laccocoris hoogstraali *La Rivers,* Heleocoris humeralis *Signoret.

Limnocorinae:* Limnocoris lutzi* La Rivers; Cryphocricinae: *Cryphocricos hungerfordi *Usinger*, Ambrysus occidentalis *La Rivers; Naucorinae: *Ilyocoris cimicoides *(Linnaeus),* Pelocoris femoratus* (Palisot de Beauvois), *Macrocoris rhantoides *Bergroth, *Naucoris maculatus* Fabricius, *Neomacrocoris handlirschi* Montandon, *Namtokocoris siamensis *Sites.

*Pleidae. Paraplea frontalis* (Fieber).

*Helotrephidae. Helotrephes semiglobosus *Stål, *Hydrotrephes visayasinensis* Zettel, *Hydrotrephes balnearius *(Bergroth), *Tiphotrephes indicus *(Distant).

*Notonectidae.* Anisopinae: *Anisops cameroonensis* Signoret, *Anisops sardea* Herrich-Schäffer, *Buenoa uhleri* Truxal; Notonectinae: *Notonecta glauca* Linnaeus, (Fabricius)*, Enithares bergrothi *Montandon,* Nychia sappho *Kirkaldy.

*Corixidae. *Corixinae:* Agraptocorixa hyalinipennis *(Fabricius),* Corixa dentipes *(Thomson), *Corixa punctata *(Illiger),* Ectemnostegella montana *Lundblad*, Hesperocorixa linnaei *(Fieber), Cymatiinae:* Cymatia coleoptrata *(Fabricius), Stenocorixinae:* Stenocorixa protrusa *Horváth.

*Diaprepocoridae. Diaprepocoris zelandiae *Hale.

*Micronectidae. Micronecta quadristrigata *Breddin.

Specimens of the Potamocoridae were not available for the purpose of the present study. 

### 2.2. Terminology Used for Descriptions of the Apical Sensilla

 With respect to the external morphology of the sensilla, in this study they are classified according to the morphological criteria established by Altner and Prillinger [6], McIver [8], and Zacharuk [7]. The receptor functions of the sensilla of the insects have been distinguished based on the morphological and ultrastructural features described by a number of authors [4, 6–8, 28, 29]. Such information is also used for the interpretation of newly described labial sensilla such as clubbed-like sensillum (CBS), paddle-like sensillum (PDS), cupola-like sensillum (CUS), finger-like sensillum (FRS), freniale-like sensillum (HLS), chaetic sensillum with a divided tip (CHD), star-like sensillum (STS), and multilobed sensillum (MPS). The remaining types of sensilla mentioned in the present paper (Table 1) are known from previous descriptions of other authors [4, 6–8, 15, 24]. Table 1 includes information about functional and morphological classifications and provides definitions of the sensilla of insects used for current descriptions of the twenty-four types of labial sensilla in the Nepomorpha. Abbreviations of sensilla used throughout the paper are explained in the last column.

## 3. Results

### 3.1. Morphology and Categories of the Labial Sensilla

Functionally, the labial sensilla are classified into two categories: mechanoreceptive and chemoreceptive sensilla, within which there can be determined twenty-four types (21 types of mechanosensilla, three types of chemosensilla) on the basis of their external appearance and location. The main external morphological characters indicating the types of sensilla are pores system (visible or not), the manner in which the sensilla are sunken with respect to the surface of the labium (flexible or inflexible sockets), and the shape of the sensilla.

The twenty-four types are grouped as follows. (1) The first group includes the chaetica sensilla (CH1, CH2, and CH3), as well as conical sensilla (COS) that are dominant types of sensilla present in all studied taxa. (2) The second group includes mechanosensitive sensilla of different shapes (squamiform, trichobothrium, paddle-like, clubbed-like, ribbon-like, chaetic with bisected tip, finger-like, freniale-like, peg, cupola, basiconica, star-like, multilobated); they are usually characteristic of individual taxa (species, subfamilies, or families). (3) The third group includes chemosensitive sensilla (trichoid, plates, papillae, and peg-in-pit sensilla), which also are found in different families or subfamilies. 

In addition, structures similar to sensilla have been reported as unidentified type (PLE).

### 3.2. Dominant Types of Sensilla

#### 3.2.1. Mechanoreceptive Sensillum Sunken in Flexible Socket (Tactile Sensillum)

**Figure 1:**
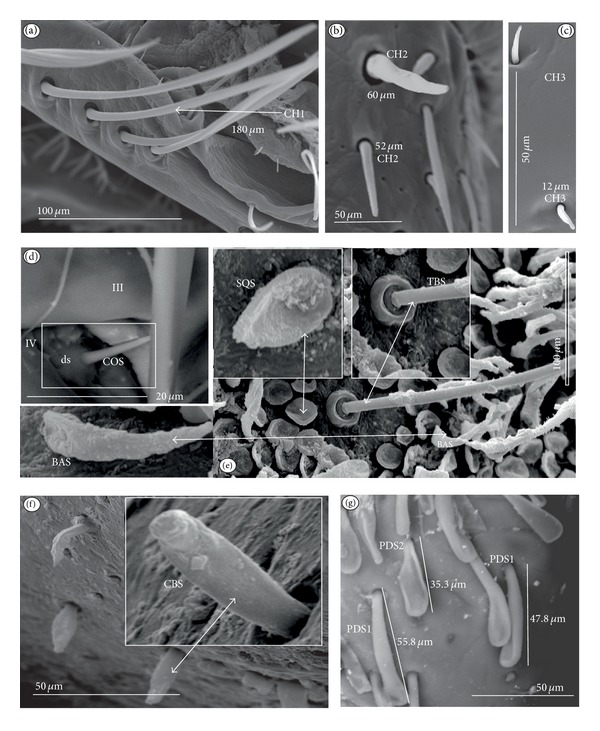
Types and sizes of the mechanosensilla of the Nepomorpha. (a) CH1 chaetic sensillum, long. (b) CH2 chaetic sensillum, medium length. (c) CH3 chaetic sensillum, short. (d) COS conical sensillum (prioprereceptive). (e) SQS squamiform sensillum, TBS trichobothrium sensillum, and BAS basiconic sensillum. (f) CBS clubbed-like sensillum. (g) PDS1 paddle-like sensillum, long, PDS2 paddle-like sensillum, short.

Chaetic sensilla (CH) (Figures 1(a), 1(b), and 1(c)) occur in different lengths and are sunken in a circular socket on the labial surface. Their external surface is usually grooved without pores. Based on the aforementioned character of morphology the three subtypes are differentiated as follows. 

*Large* (100 *μ*m and above) chaetic sensilla (CH1). The sensilla are long, relatively straight, gradually tapering and slightly curved at the tip (Figure 1(a)). 

*Medium length* (50–99 *μ*m) chaetic sensilla (CH2). Shorter than (CH1) with a fine tip and a strong base (Figure 1(b)).

*Short *(1–49 *μ*m) chaetic sensilla (CH3) with sharp ends (Figure 1(c)). 

The chaetic sensilla are present in all examined species in various parts of the labium.

*Conical Sensillum *(COS): a Prioprereceptor (Figure 1(d)). This sensillum is a short or long cone with a smooth surface. It is sunken in a dome-shaped socket (ds).

### 3.3. Characteristic Forms of the Mechanosensilla

*Squamiform sensillum* (SQS) (Figure 1(e)). This sensillum is slightly rhombic-shaped and has been found in the Nepinae (*Laccotrephes japonensis,* Figures 9(d) and 9(f)) as well as in *Curicta granulosa*, *Borborophyes mayri,* and *Nepa cinerea *(Table 2).

*Trichobothrium sensillum* (TBS) (Figure 1(e)). A hair (“trich” = hair) is long and tapering at the end. Its basal part is sunken in a socket. The flexible socket (Soc) is rounded and is placed on a dome-like cuticular elevation (= bothrium (bt)). The hair is oriented at a more or less right angle to the cuticle. The cuticular surface surrounding the trichobothrium is usually devoid of other sensilla. Several trichobothria have been observed only in the Nepinae (*Laccotrephes japonensis,* Figures 9(d) and 9(f)), as well as in *Curicta granulosa*, *Borborophyes mayri* and *Nepa cinerea*.

*Basiconic sensillum* (BAS) (Figure 1(e)). The sensillum is tapered gradually from a wide base to the tip and is relatively stiff. This sensillum has been found in the Nepinae (*Laccotrephes japonensis* (Figure 9(d)) as well as in *Curicta granulosa*, *Borborophyes mayri,* and *Nepa cinerea*.

*Clubbed-like sensillum* (CBS) (Figure 1(f)). In this sensillum the base and shaft have the same width and the tip is slightly rounded. This type of sensillum has been noticed in the Nepinae (*Laccotrephes japonensis* (Figures 9(a), 9(b), and 9(c)) as well as in *Curicta granulosa*, *Borborophyes mayri,* and *Nepa cinerea.*

*Paddle-like sensillum* (PDS) (Figure 1(g)). This sensillum is narrow in its lower part, then gradually expands up to a wide, flattened tip. Two sizes of this sensillum are distinguished (Figure 1(g)): a long PDS1 (47–55 *μ*m) and a short PDS2 (35 *μ*m). These sensilla have been observed in the Ranatrinae: *Ranatra chinensis,* Figures 10(a), 10(b), 10(d), and 10(f). 

**Figure 2:**
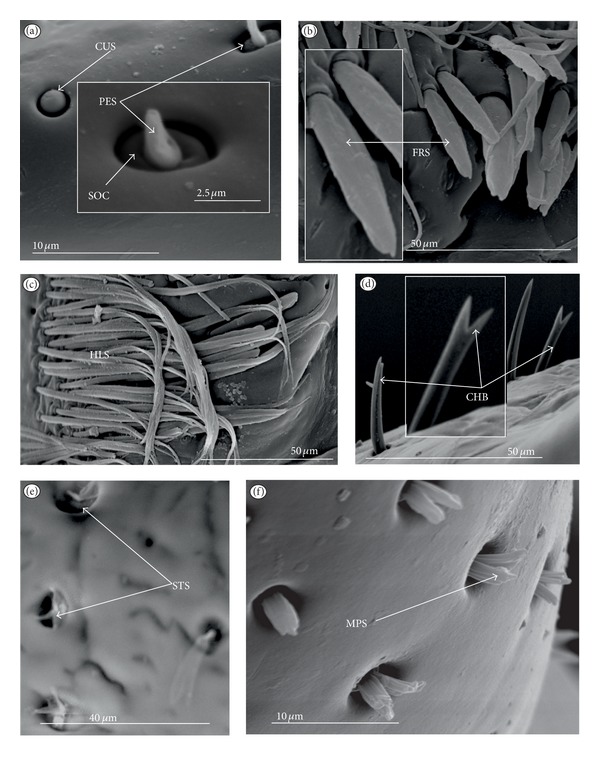
Types and sizes of the labial mechanosensilla of the Nepomorpha. (a) CUS cupola sensillum, PES peg sensillum. (b) FRS finger-like sensillum. (c) HLS freniale-like sensillum. (d) CHB chaetic sensillum with a bisected tip. (e) STS star-like sensillum. (f) MPS multilobed sensillum.

*Cupola-shaped sensillum* (CUS) (Figure 2(a)). This sensillum is short and protrudes slightly above the surface of the labium. This type has been identified in the Belostomatidae (*Belostoma flumineum,* Figures 11(b) and 11(c); *Hydrocyrius colombiae *Figures 12(a) and 12(b); *Lethocerus deyrollei,* Figures 14(b) and 14(c)) and *Appasus major* (Table 2). It has also been observed in the Ochteridae (*Ochterus piliferus,* Figures 15(b) and 15(c): *O. marginatus,* Figure 15(f)), Gelastocoridae (*Gelastocoris oculatus,* Figures 16(a) and 16(b); *Nerthra nepaeformis,* Figures 17(a), 17(b), 17(c), and 17(d)) and the Aphelocheiridae (*Aphelocheirus aestivalis,* Figures 18(a) and 18(b)).

*Peg sensillum* (PES) (Figure 2(a)). It is a short cone sunken in a shallow cavity of cuticle and equipped with a flexible socket (SOC). This type of sensillum has been found in the Belostomatidae (*Belostoma flumineum* Figure 11(c); *Hydrocyrius colombiae,* Figures 12(a) and 12(b); *Limnogeton fieberi,* Figure 13(a); *Lethocerus deyrollei*, Figures 14(b) and 14(c)) and *Appasus major* (Table 2) as well as in the Ochteridae (*Ochterus piliferus,* Figures 15(b) and 15(c); *O. marginatus,* Figure 15(f)); Gelastocoridae (*Gelastocoris oculatus* Figures 16(a) and 16(b), *Nerthra nepaeformis,* Figures 17(a), 17(b), 17(c) and 17(d)), Aphelocheiridae (*Aphelocheirus aestivalis,* Figures 18(a) and 18(b)) and in some Naucoridae; in the Cheirochelinae (*Cheirochela feana*, Figures 19(a) and 19(b)), (*Gestroiella limnocoroides*, Figure 19(f)), and *Coptocatus kinabalu*, *Coptocatus oblongulus*, *Tanycricos longiceps* (Table 2), Laccocorinae (*Laccocoris hoogstraali*, Figures 20(a) and 20(b); *Helocoris humeralis*, Figures 20(e) and 20(f)), Corixidae, (*Corixa dentipes,* Figure 29(d), *Cymatia coleoptrata,* Figures 30(b), 30(e), and 30(f)), and *Hesperocorixa linnaei, Ectemnostegella montana, Agraptocorixa hyalinipennis, Corixa punctata, Stenocorixa protrusa,* Table 2), Diaprepocoridae, (*Diaprepocoris zealandiae,* Figures 31(a) and 31(b)) and finally in the Micronectidae (*Micronecta quadristrigata,* Figure 31(e)).

*Finger-like sensillum* (FRS) (Figure 2(b)). The base and tip of this type of sensillum are of the same width, but the shaft is slightly wider in the middle. This type of sensilla has been observed only in the Gelastocorinae (*Gelastocoris oculatus* Figure 16(f)).

*Freniale-like sensillum* (HLS) (Figure 2(c)). This sensillum is designed as a long, thin hair with a tapered tip. It has been observed in the Gelastocorinae (*Gelastocoris oculatus,* Figures 16(b), 16(f), and 16(g)).

*Chaetic sensillum with a bisected tip *(CHB) (Figure 2(d)). The tip of the seta is divided into two short branches. This type of sensillum has been found only in the *Nerthra nepaeformis* (Figure 17(e)) (Gelastocoridae: Nerthrinae).

*Star-like sensillum* (STS) (Figure 2(e)). It is a short cone divided into four or five narrow lobes. The base of the sensillum is sunken in a socket, and it is situated shallowly in a cavity. The lobes are prominent above the cuticular surface. This type of sensillum has been specific to the Aphelocheiridae (*Aphelocheirus aestivalis,* Figures 18(c), 18(d), and 18(e) and *A. variegatus*).

*Multilobed sensillum *(MPS) (Figure 2(f)). This type of sensillum consists of a few narrow lobes, arising from a common stem. The base of the sensillum is sunken in a socket and the lobes evidently protrude above the cuticular surface. This type of sensillum has been found in the Limnocorinae (*Limnocoris lutzi*, Figures 21(a) and 21(c)), Cryphocricinae (*Cryphocricos hungerfordi*, Figures 22(a), 22(b), 22(c), and 22(d); *Ambrysus occidentalis,* Figures 23(a), 23(c) and 23(e)) and the Naucorinae (*Naucoris maculatus*, Figure 24(a); *Namtokocoris siamensis,* Figure 24(d); *Neomacrocoris handlirschi,* Figure 24(g)) as well as in *Ilyocoris cimicoides* and *Pelocoris femoratus, *(Table 2).

**Figure 3:**
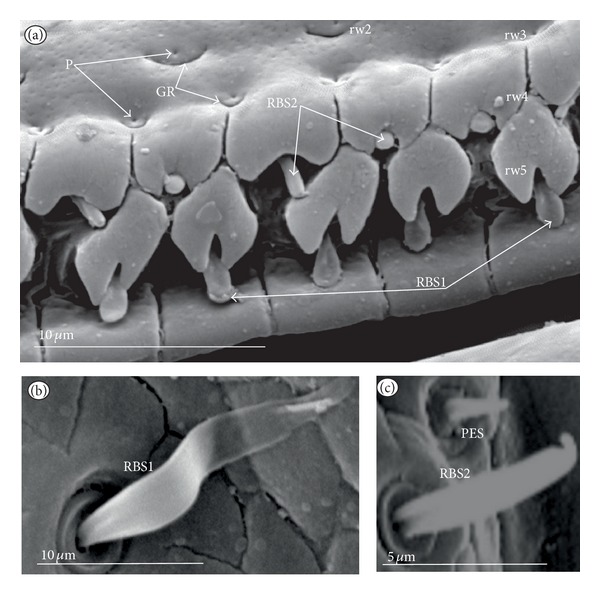
Types and sizes of the labial sensilla of the Nepomorpha (Corixoidea). (a) RBS ribbon-like sensillum. (b) RBS1 ribbon-like sensillum, long. (c) RBS2 ribbon-like sensillum, short, PES peg sensillum. g: groove, p: pore, rw1, rw2: the first and second row of semicircular grooves, rw3: the third row with the pores, rw4, rw5: the fourth and fifth rows with the ribbon-like sensillum.

*Ribbon-like sensillum* (RBS) (Figures 3(a), 3(b), and 3(c)). The shaft of this sensillum is a long, wide, and flexible lobe with a blunt ending. The base is slightly narrower than the shaft and is sunken in the socket. These sensilla have different lengths. A long sensillum of this type is 7.5 *μ*m (RBS1) whereas its short counterpart (RBS2) is 4.0 *μ*m. Both types of sensilla are numerous and have been found in the Corixidae, (*Corixa dentipes,* Figure 29(c); *Cymatia coleoptrata,* Figures 30(b), 30(e), and 30(f)); and* Hesperocorixa linnaei, Ectemnostegella montana, Agraptocorixa hyalinipennis, Corixa punctata, Stenocorixa protrusa,* Table 2), Diaprepocoridae, (*Diaprepocoris zealandiae,* Figures 31(a) and 31(b)) and Micronectidae (*Micronecta quadristrigata,* Figure 31(e)).

The unidentified type (PLE) has been found only in *Limnocoris lutzi* (Figure 21, Limnocorinae) and *Cryphocricos hungerfordi* (Figure 22, Cryphocricinae). These structures are small plates with several peg-like endings. They are distributed on the surfaces of the first, second, and third segment, but not on the fourth segment of the labium.

### 3.4. Chemosensitive Sensilla and Their Distributions

#### 3.4.1. Contact-Chemoreceptive Sensillum Sunken in a Flexible Socket

**Figure 4:**
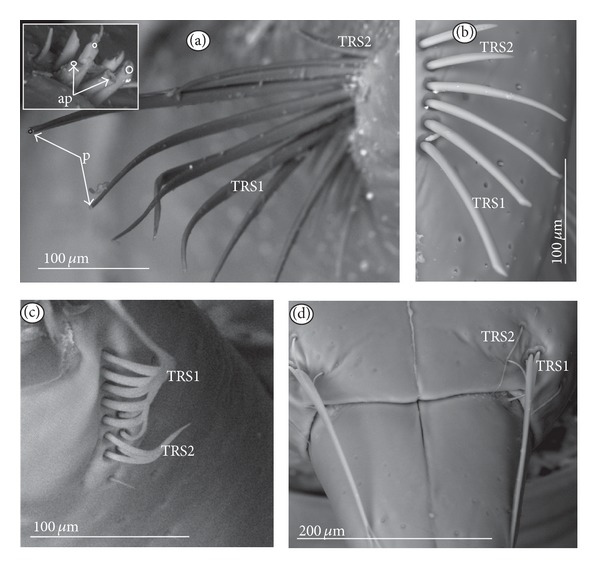
Types and sizes of the trichoid sensilla of the Nepomorpha. (a) TRS1 trichoid sensillum, long, TRS2 trichoid sensillum, short; these sensilla are placed dorsally near the apex of the labium at the fourth segment; in the broken shank of the sensillum, a hole (ap) is visible, and it displays a dendrite running to the tip of the sensillum. (b) TRS1, TRS2 trichoid sensilla; these are individually placed dorsally near the apex of the labium in one row. (c) TRS1, TRS2 trichoid sensilla; these are placed ventrally, near the apex of the labium. (d) TRS1, TRS2 trichoid sensilla; they are placed dorsally on the third labial segment.

*Trichoid sensillum* is a contact chemoreceptive sensillum (TRS) (Figures 4(a)–4(d)). Trichoid sensilla are usually strong setae placed in shallow depressions in flexible sockets. They are often slightly curved just above their bases and protrude at low angles (about 20° in the case of a dorsal and 50° in the case of a ventral trichoid sensillum) from the base towards labial tip (Figures 4(a), 4(b), and 4(c)). They have a smooth surface and are tapered distally. The terminal pore is visible and therefore these sensilla should be considered as contact-chemoreceptive sensilla; this is also suggested by their location. Trichoid sensilla (TRS) are, respectively, subdivided into two groups according to their size (length; large TRS1, short TRS2) and into three groups according to their location.

*Large* (50–100 *μ*m) (TRS1) and *short* (1.0–49 *μ*m) (TRS2) trichoid sensilla are placed on the *dorsal side on the fourth segment* of the labium (Figures 4(a) and 4(b)). These sensilla have been identified in the Nepidae (Figures 9(a) and 10(b)), Belostomatidae (Figures 11(a), 12, 13(b), 13(c), 14(a), and 14(b)), Pleidae (Figures 25(a) and 25(c)), Helotrephidae (Figures 26(a) and 26(b)), and Notonectidae (Figures 27(b), 27(d), and 28(b)).

*Large* (50–100 *μ*m) (TRS1) and *short* (1.0–49 *μ*m) (TRS2) trichoid sensilla are placed on the *ventral side on the fourth segment* of the labium (Figure 4(c)). These sensilla have been identified in the Nepidae (Figures 9(b), 10(b), and 10(c)), Belostomatidae (Figures 11(b), 12(e), 13(c), and 14(b)), Ochteridae (Figures 15(c) and 15(g)), Gelastocoridae (Figures 16(d) and 17(b)), Aphelocheiridae (Figure 18(b)), Naucoridae, Cheirochelinae (Figures 19(b) and 19(g)), Laccocorinae (Figure 20(f)), Limnocorinae (Figure 21(c)), Cryphocricinae (Figures 22(a) and 23(d)), Naucorinae (Figures 24(a), 24(b), 24(d), 24(e), and 24(g)), Pleidae (Figure 25(b)), Helotrephidae (Figure 26(e)), and Notonectidae (Figures 27(e) and 28(e)).

*Large* (50–100 *μ*m) (TRS1) and *short* (1.0–49 *μ*m) (TRS2) trichoid sensilla placed on the *dorsal side on the third segment* of the labium (Figures 4(a) and 4(b)). These sensilla have been identified only in the Naucoridae, Cheirochelinae (Figures 19(a), 19(d), 19(f), and 19(h)), Laccocorinae (Figures 20(a), 20(b), 20(e), and 20(f)), Limnocorinae (Figure 21(e)), Cryphocricinae (Figure 23(c)), and Naucorinae (Figures 24(a), 24(b), 24(d), and 24(f)).

### 3.5. Chemoreceptive Sensillum Sunken in an Inflexible Socket

**Figure 5:**
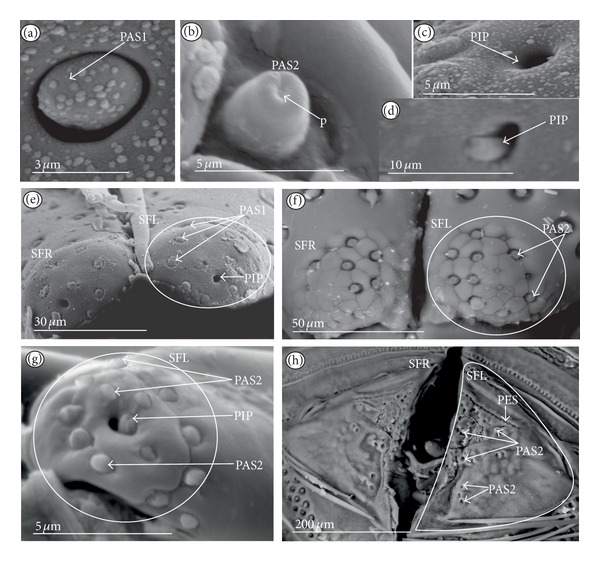
Types and arrangements of the labial apical sensilla of the Nepomorpha. (a) PAS1 papilla-like sensillum, flattened. (b) PAS2 papilla-like sensillum, rounded. (c) PIP peg-in-pit sensillum, only the pit is visible. (d) Peg-in-pit sensillum, the peg is visible. (e) PAS1 sensilla are circularly placed on the smooth, rounded labial tip with a slightly lateral position of the PIP. These are arranged symmetrically on the right (SFR) and left (SFL) sensory field. (f) PAS2 sensilla are circularly placed on the folded labial tip. The PIP are invisible. They are arranged symmetrically on the right (SFR) and left (SFL) sensory fields. (g) PAS2 sensilla are circularly placed on the labial tip with the central position of the PIP sensillum; the left (SFL) sensory field is shown. (h) PAS2 sensilla are irregularly placed on the labial tip. These sensilla are arranged symmetrically on the right (SFR) and left (SFL) sensory fields. PES form a short row; RBS ribbon-like sensilla (mechanosensilla) are not numerous.

*Papilla sensillum*—gustative sensillum (PAS) (Figures 5(a) and 5(b)). This type of sensillum is wide and low. Two subtypes have been recognized as PAS1 (Figure 5(a)) with a flattened tip and PAS2 (Figure 5(b)) with a slightly rounded tip. They possess inflexible sockets and are placed in a depression of the cuticle. On the tip of this type of a sensillum there is visible a terminal pore, and therefore these sensilla should be considered as gustatory chemoreceptive sensilla; it is additionally suggested by their location. Papillae sensilla are distributed only over the labial tip (Figures 5(e)–5(h)).

The papillae sensilla (PAS1) have been found in the Nepidae (Figures 5(a), 5(b), 9(e), and 10(g)), Belostomatidae (Figures 11(f) and 14(f)), and Nerthrinae (Gelastocoridae Figure 17(g)). The papillae sensilla PAS2 have been observed in the Ochteridae (Figure 15(g)), Gelastocorinae (Figure 16(h)), Aphelocheiridae (Figure 18(g)), Cheirochelinae (Figures 19(b) and 19(i)), Laccocorinae (Figure 20(d)), Limnocorinae (Figure 21(b)), Cryphocricinae (Figures 22(e), and 23(e)), Naucorinae (Figure 24(e)), Pleidae (Figure 25(f)), Helotrephidae (Figure 26(f)), Anisopinae (Figure 27(d)), Notonectinae (Figure 28(f)), Corixinae (Figure 29(e)), Diaprepocoridae (Figures 31(a) and 31(b)), and Micronectidae (Figure 31(e)).

*Peg-in-pit sensillum* (PIP) is a thermohygroreceptive sensillum (Figures 5(c) and 5(d)). This type of sensillum has a small peg inserted in a round deep depression. The walls of the sensillum are smooth without pores. One such sensillum is present on the lobes (sensory fields) (Figure 5(g)) and usually is situated either centrally or more laterally; however, it is not always visible in some studied species. Generally, this type of sensillum is observed frequently in the species of the Nepidae (Figure 9(e)), Belostomatidae (Figure 11(f)), and Nerthrinae (Figure 17(g)) which are characterized by the smooth tip of the labium. In the remaining groups the labial tip is divided into a few furrows, which probably in most species is hiding this type of sensillum, which is, however, visible in the Pleidae (*Paraplea frontalis,* Figure 25(f)), Helotrephidae (*Hydrotrephes visayasinensis,* Figure 26(f)), and Notonectinae (*Notonecta glauca,* Figure 28(f) sensillum no 6). Sensilla of this type are distributed only over the labial tip.

### 3.6. Organization of the Labium

In most of the aquatic bugs, the elongated labium is divided into four segments (I, II, III, and IV) except for in the Corixidae, Diaprepocoridae, and the Micronectidae. In the latter families the labium is short and wide, and the segmentation of the labium is not visible. The tip of the labium (one half) is slightly triangular with a clearly wrinkled (folded) surface. In the remaining nepomorphan families, the labium at the tip becomes slightly rounded to form two lateral lobes (left-LL and right-LR) and a ventral (V) one, that is, apical plate (Figures 5(c) and 5(d)). At the tips of both lateral lobes there are sensory fields (SFR—right and SFL—left) (Figures 5(e)–5(h)) including various morphological types of sensilla: papilla (Figures 5(a) and 5(b)), peg-in-pit sensilla (Figures 5(c) and 5(d)), ribbon-like sensilla, and peg sensilla. The most of the labial tip sensilla are embedded into inflexible sockets set deeply between folds. The apical surface of the lateral lobes is smooth in the Nepidae and the Belostomatidae (Figure 5(e)), and in other families the surface is divided by deep furrows into several folds (Figure 5(f)).

### 3.7. The Distribution of Apical Chemosensilla in the Systematic Groups

**Figure 6:**
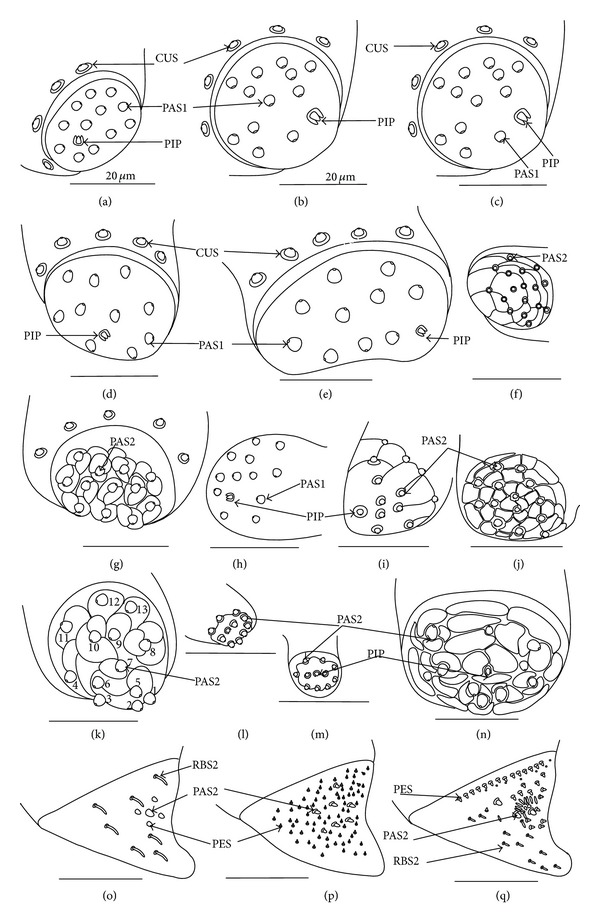
Types of distribution of the labial apical sensilla in the Nepomorpha: Type A Nepidae, Belostomatidae, and Nerthrinae. (a) *Nepa cinerea*. (b) *Laccotrephes japonensis*. (c) *Ranatra chinensis*. (d) Belostomatinae; *Appasus major*. (e) Lethocerinae; *Lethocerus deyrollei*. (f) Type B, Ochteridae; *Ochterus marginatus*. (g) Type C, Gelastocorinae; *Gelastocoris oculatus*. (h) Type A, Nerthrinae; *Nerthra nepaeformis*. (i) Type B, Aphelocheiridae; *Aphelocheirus aestivalis*. (j) Type C, Cheirochelinae; *Coptocatus oblongulus*. (k) Limnocorinae; *Limnocoris lutzi.* (l) Helotrephidae; *Helotrephes semiglobosus*. (m) Type D, Pleidae; *Paraplea frontalis*. (n) Type C, Notonectinae; *Notonecta glauca*. (o) Type E, Cymatiinae; *Cymatia coleoptrata*. (p) Diaprepocoridae; *Diaprepocoris zealandiae*. (q) Micronectidae; *Micronecta quadristrigata*. PAS1 papilla sensillum, flattened; PAS2 papilla sensillum, rounded; PIP peg-in-pit sensillum; CUS cupola-like sensillum located outside the labial tip; RBS ribbon-like sensillum; PES peg sensillum.

Five patterns of distribution of the apical sensilla have been identified (Figures 6(a)–6(q)) as follows.

- The pit sensilla (one pair) are localised more laterally, and the papillae sensilla (PAS1) are distributed over the rounded tip of the labium. The number of sensilla (PAS1) ranges from 10 to 13 pairs in the Belostomatidae and Nepidae (Figures 6(a)–6(e)).
- The papillae sensilla (PAS2) are present in the number of 11-12 pairs. On the surface of the labial tip there are shallow furrows. The PIP sensilla are invisible. This type of the distribution of these sensilla is characteristic for the Ochteridae and Aphelocheiridae (Figures 6(f) and 6(i)).
- The papillae sensilla (PAS2) are present in the number of 8–14 pairs. They are sunken in the cuticle, and folds are formed around them. The PIP sensilla are not evident; however, in some species they are visible (Figure 24(e)). This type of distribution has been observed in the Gelastocoridae (except the Nerthrinae where the tip is smooth; (Figure 6(h))), Naucoridae, and Notonectidae (Figures 6(g), 6(j), 6(k), and 6(n)).
- One pair of pit sensilla is placed centrally together with two PAS2 on the round convex surface, while the remaining papillae sensilla (PAS2) in the number ranging from 8 to 11 pairs are distributed around them. This pattern has been found in the Pleidae and Helotrephidae (Figures 6(l) and 6(m)).
- The papillae sensilla PAS2 are more numerous and visible on the triangular labial tip. They are spread unevenly in cuticular folds. This type of distribution of the sensilla is specific to the Corixidae and Micronectidae. Only in the Diaprepocoridae the sensilla (PAS2 and PES) are sunken deep in the smooth surface of the tip (Figures 6(o), 6(p), and 6(q)). The peg sensilla (PES) and ribbon-like sensilla (RBS2), however, are present at the labial tip; these are mechanosensilla.

### 3.8. Distribution Types of the Mechnosensilla and Contact-Chemoreceptive Sensilla on the Labial Segments in the Systematic Groups

The presence or absence of different types of mechanosensilla on the labium in the 55 species is presented in Table 2. A distinction of general types of distributions of the mechanosensilla in taxonomic groups is as follows.

#### 3.8.1. Sensilla Numerous, Grouped, and Unevenly Arranged on the Labium (Figure 7(a))

*The Belostomatidae*. On the labial segments (I, II, and III) the sensilla chaetica (CH1, CH2, and CH3) are numerous and placed in groups or rows distributed along the labium near the labial groove on the dorsal side; apart from that several chaetica, sensilla are unevenly scattered on the surface of those segments (Figure 11(e)). On the IV segment the sensilla (CUS and PES) are numerous and unevenly scattered. Similar distribution can be found on the ventral side with less numerous sensilla. More differences in distribution of the sensilla can be observed in *Limnogeton fieberi* (Figure 13). The II and III segment on the dorsal and ventral sides are densely covered by short sensilla CH3; however, on the dorsal side near the labial groove sensilla CH1 and CH2 are also numerous. These sensilla (CH1, CH2 and CH3) are placed dorsally and form a dense layer. Trichoid sensilla (two dorsal pairs and three ventral pairs) are similarly placed in both subfamilies (Belostomatinae and Lethocerinae), subapically on the IV segment but in various numbers and size (Table 2).

#### 3.8.2. Sensilla Densely and Evenly Arranged on Labium (Figures 7(b), 7(c), and 7(d))

*The Nepinae.* On the labial segments (II, III: I—the first is invisible) the sensilla (SQS) are numerous and totally cover the surface of the segments to form a main layer with several of the BAS, TBS, and CH1 sensilla. On the IV segment the sensilla (CBS) are less numerous and are evenly scattered. 

*The Ranatrinae.* On the labial segments (II, III, IV; I—the first is invisible) the sensilla (PDS1, PDS2) are not numerous and form the main layer. Among the PDS1 and PDS2 several chaetica sensilla (CH1, CH2) are visible. On the IV segment the sensilla (CH1, CH2, and CH3) are unevenly scattered and less numerous. Trichoid sensilla (three pairs dorsal, lateral, and ventral) are similarly placed in both subfamilies (Nepinae and Ranatrinae), subapically on the IV segment but in various numbers and sizes.

*The Ochteridae.* On the labial segments (I, II, III) the sensilla (CH1, CH2, and CH3) are numerous. On the IV segment the sensilla (CUS and PES) are numerous and situated in regular rows. Trichoid sensilla (one pair of TRS2) are placed ventrally and subapically on the IV segment.

*The Gelastocorinae.* On the labial segments (I) and (II) sensilla (HLS, CH2) and sensilla (CH2, HLS, and FRS), respectively, are numerous and cover the whole surface. On the III segment sensilla (CH1, CH2) generally are not numerous and are placed rather laterally while the CH3 are numerous. On the IV segment the sensilla (CUS and PES) are numerous and are situated in regular rows. Trichoid sensilla (three pairs of TRS2) are placed ventrally and subapically on the IV segment.

*The Nerthrinae*. On the labial segments (I, II, III) the sensilla (CH2, CH3) are numerous. On the III segment the sensilla (CHB, CUS, and PES) are also numerous. On the IV segment sensilla (CUS and PES) are numerous and situated in regular rows. Trichoid sensilla (one pairs TRS2) are placed ventrally and subapically on the IV segment.

#### 3.8.3. Sensilla Less Numerous and Numerous Evenly Arranged (Figures 7(e), 7(f), and 7(g))

*The Aphelocheiridae.* On the labial segments (I, II, and III) the sensilla (CH2, CH3) are not numerous. On the III segment the sensilla STS and PES are numerous whereas on the IV segment the sensilla CUS and PES are numerous; they are situated in regular rows. Trichoid sensilla (one pair of TRS2) are placed subapically on the IV segment.

*The Cheirochelinae and Laccocorinae.* On the labial segments (I, II, and III) the sensilla (CH2, CH3) are not numerous. On the IV segment the sensilla (PES) are numerous and densely cover this segment; the sensilla are less numerous on the III segment. In different species (Table 2), from 2 to 8 TRS1 and TRS 2 sensilla are distributed at the distal edge of the III segment and from 5 to 10 are ventrally distributed on the IV segment.

**Figure 7:**
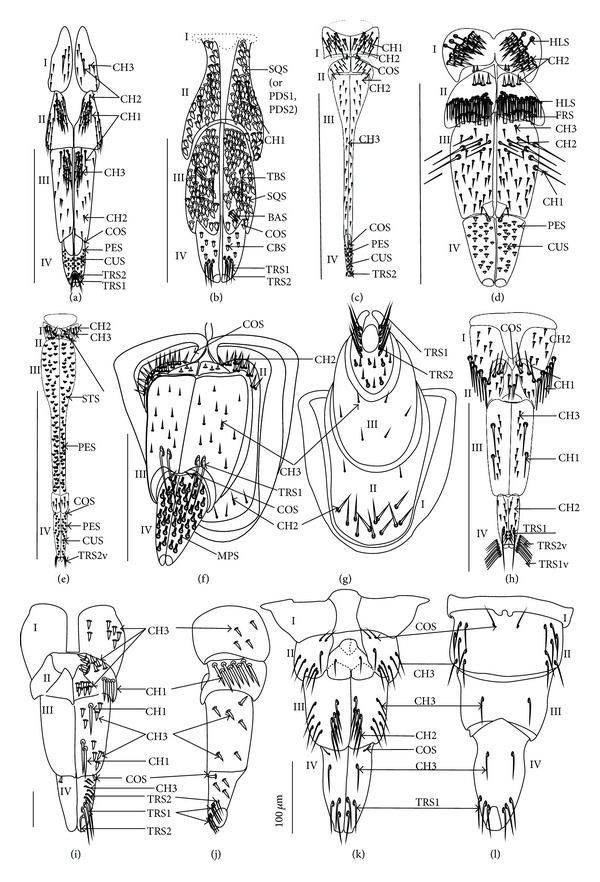
Types of distribution of the mechanosensilla on the labial segments in the Nepomorpha (except for the Corixoidea). Sensilla numerous, grouped, and unevenly arranged on the labium ((a) Belostomatidae). Sensilla densely and evenly arranged on the labium ((b) Nepinae, Ranatrinae: I segment reduced dorsally); ((c) Ochteridae); ((d) Gelastocorinae). Sensilla less numerous and numerous evenly arranged ((e) Aphelocheiridae); ((f) dorsal side (g) ventral side of Cheirochelinae, Laccocorinae, Limnocorinae, Cryphocricinae, and Naucorinae). Sensilla are not very numerous and scattered unevenly ((h) Notonectinae); ((i) dorsal side, (j) lateral side of Pleidae); ((k) dorsal side, (l) ventral side of Helotrephidae).

**Figure 9:**
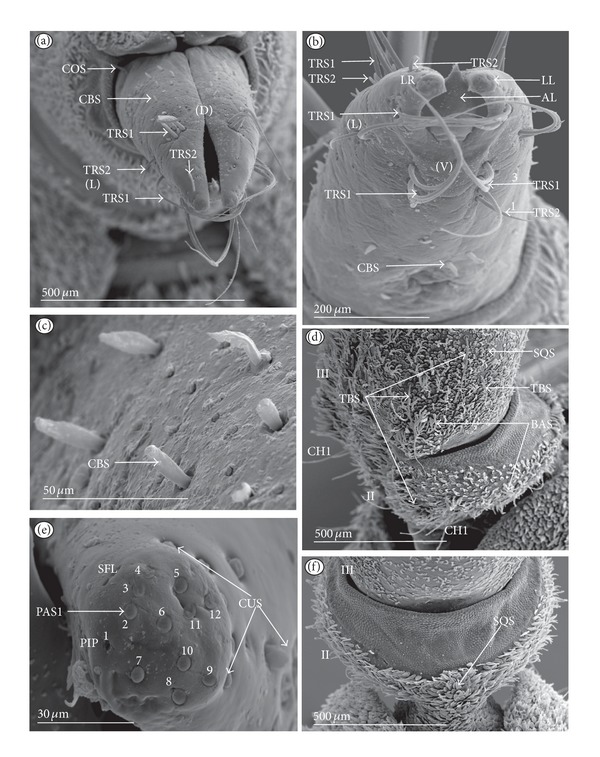
Types and sizes of sensilla of the Nepinae (*Laccotrephes japonensis*). (a) TRS1, TRS2 are placed dorsally (D) near of apex on the last segment of the labium, and TRS1, TRS2 are distributed laterally (L), CBS are distributed all over the surface of the last segment of the labium, and COS are visible in the proximal part of the IV segment. (b) TRS 1, TRS2 are placed ventrally (V) near the apex of the labium. LL: lateral left lobe, LR: lateral right lobe, Al, apical plate. (c) CBS are numerous and evenly distributed. (d) TBS, BAS, SQS, and CH1 are located dorsally and ventrally on III and II labial segment. (e) PAS1 (no. 2–12) and PIP (no. 1). (f) SQS are densely distributed over the ventral side.

**Figure 10:**
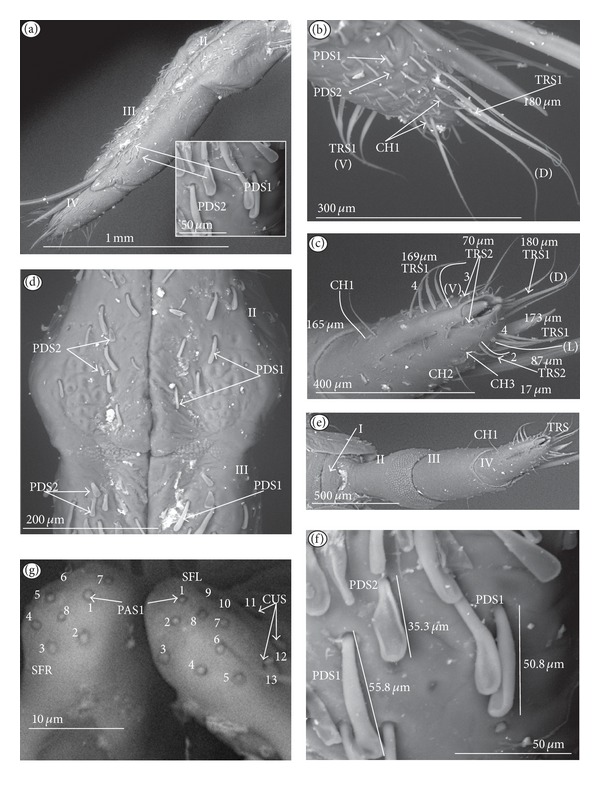
Types, sizes and distribution of the sensilla of the Ranatrinae (*Ranatra chinensis*). (a) PDS1, PDS2, CH1, and CH2 are irregularly distributed on the segment II, III, and IV, dorsal view. (b) TRS1 are placed dorsally (D) near the apex of the labium (fourth segment). (c) TRS1, TRS2 distributed laterally (L), and ventrally (V); CH1 and CH2 are distributed all over the surface of the last segment of the labium. (d) PDS1, PDS2 evenly distributed, dorsal view of the II and III segment. (e) Ventral view of the I, II, and III segments, sensilla are not very numerous; they seem more numerous on the IV labial segment. (f) Shapes and sizes of the PDS1 and PDS2. (g) PASI (no 1–10) distributed apically, PIP is invisible.

**Figure 11:**
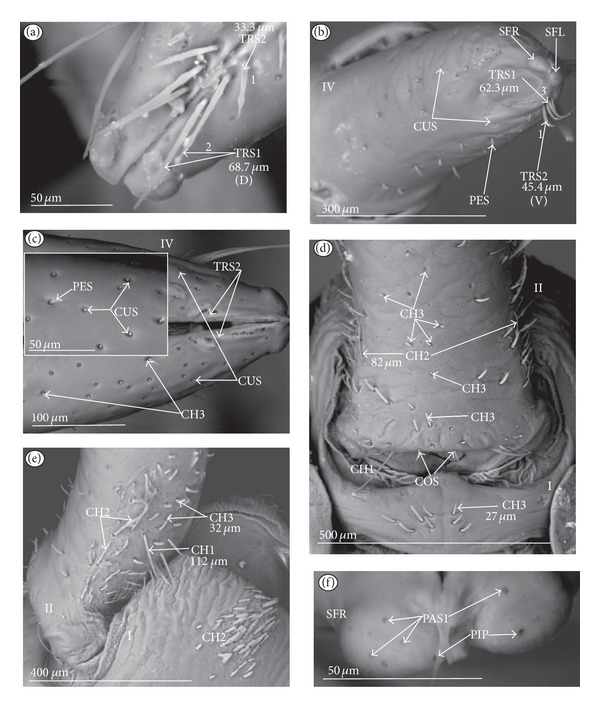
Types, sizes, and distribution of the sensilla of the Belostomatinae (*Belostoma flumineum*). (a) TRS l, TRS2 are placed dorsally (D) near the apex of the labium. (b) TRS1, TRS2 are distributed ventrally (V); CUS, CH2 are distributed sparsely and unevenly all over the surface of the last segment of labium. (c) CUS and PES are placed dorsally; they are numerous and unevenly distributed over segment IV. (d) CH1, CH2, and CH3 sensilla are sparsely and unevenly distributed, ventral view on the I and II segments are one pair of the COS is situated ventrally; on the border between the I and II segments. (e) Lateral view of the I and II segment; sensilla are densely distributed ventrally, on segment I can be seen one group of the CH1 situated dorsally. (f) PAS1 (no. 1–10), PIP (no. 11) are distributed apicaily on the sensory fields: right (SFR) and left (SFL).

**Figure 12:**
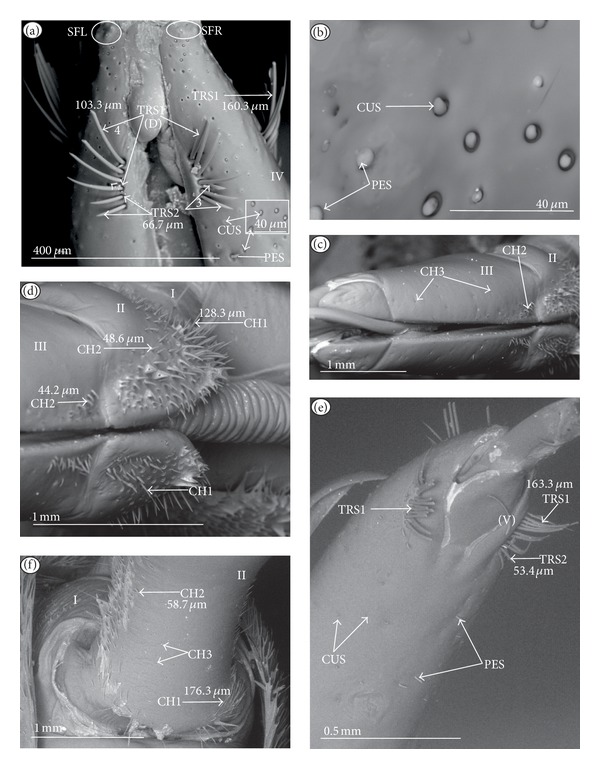
Types, sizes, and distribution of the sensilla of the Belostomatinae (*Hydrocyrius colombiae*). (a) TRS1 (four), TRS2 (three) form one row and are placed dorsally (D) near the apex of the labium; sensory fields (SFR, SFL); CUS and PES, placed dorsally, are numerous and unevenly distributed on segment IV. (b) CUS and PES (magnified). (c) CH3 are sparsely and unevenly distributed, dorsal view of segment III, several CH2 grouped near the labial groove. (d) CH2 are numerous, placed on the dorsal side, on segment II. (e) TRS1, TRS2 are distributed ventrally (V); CUS, PES, and CH3 are sparsely and unevenly distributed ventrally on the IV segment. (f) CH1 are visible at the base of segment II on the ventral side, CH2 are densely arranged along the surface of the second segment, and CH3 are slightly visible and unevenly distributed over segment II.

**Figure 13:**
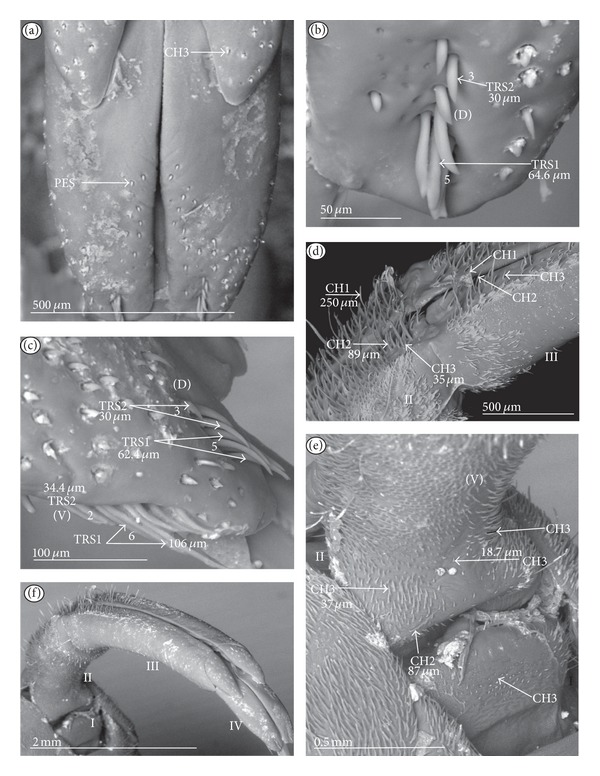
Types, sizes, and distribution of the sensilla of the Belostomatinae (*Limnogeton fieberi*). (a) PES are specifically situated only in the small area near the apex. (b) TRS2 (three), TRS1 (five) form a single row and are placed dorsally (D) near the apex of the labium. (c) TRS2 (two), TRS1 (six) form a single row and are placed ventrally (V) near the apex of the labium. (d) CH1, CH2, and CH3 densely cover the dorsal surface of the labium on segments II and III. (e) CH3 are numerous and is cover all surface of the ventral side of segment II, one pair of CH2 and at the base of the second segment, CH3 are densely distributed over segment I (ventrally). (f) Total view on the labial segments showing type (A) distribution of the labial sensilla.

**Figure 14:**
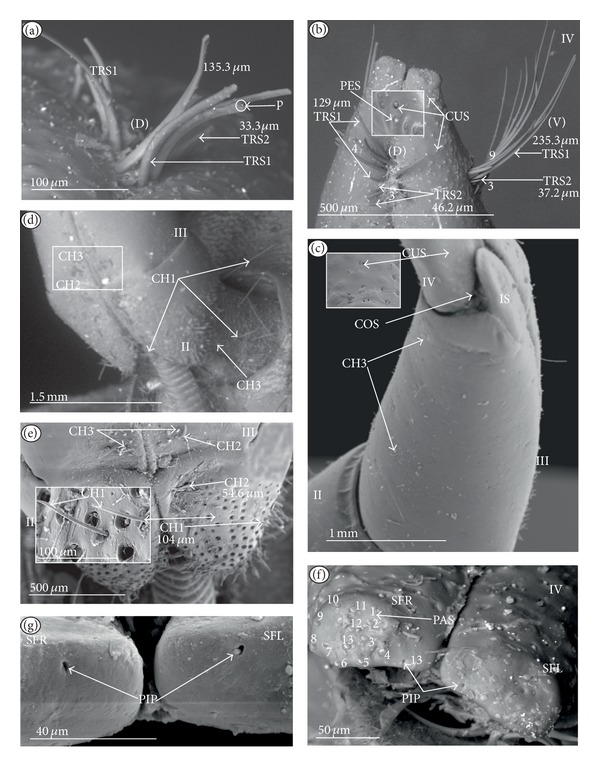
Types, sizes, and distribution of the sensilla of the Belostomatinae (*Lethocerus deyrollei*). (a) TRS2 and TRS1 are placed dorsally (D) near the apex of the labium; the pore (ap) is visible at the end of TRS1. (b) TRS2 (three), TRS1 (four) form a single row dorsally (D), TRS2 (three) and TRS1 (nine) form a tuft ventrally (V), and CUS and PES are numerous on the IV segment. (c) CH3 are spread sparsely on III segment, and COS is situated on segment IV and hidden under IS (intercalary sclerites) of the III segment. (d) CH1, CH2, and CH3 densely cover the dorsal and ventral surfaces of the labium on segments II and III. (e) CH3 and CH2 are less numerous and placed dorsally in a small group on segment III. CH1 are numerous on the dorsal surface of the II segment. (f) PAS1 (no. 1–13) and PIP (no. 14) are distributed apically over sensory fields (SFR, SFL). (g) PIP situated in the lateral positions on SRF and SFR are clearly visible.

**Figure 15:**
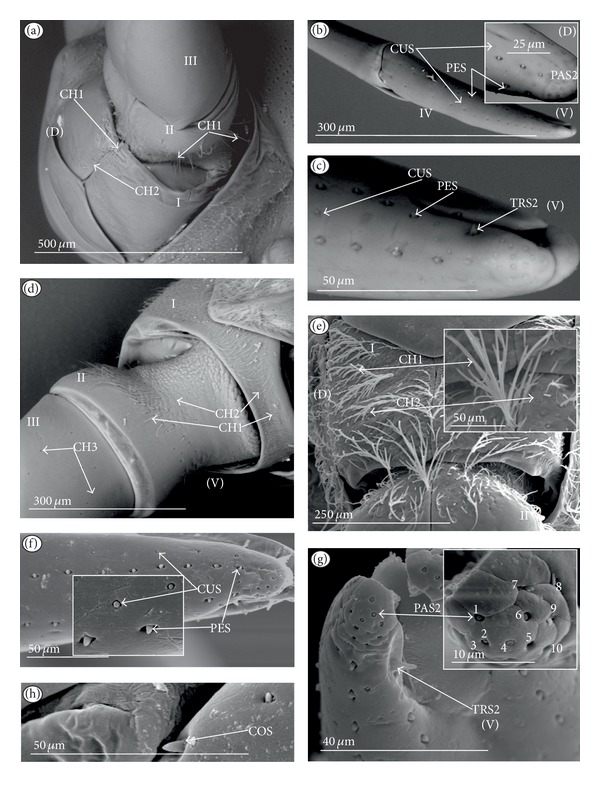
Types and sizes of the sensilla of the Ochteridae ((a)–(d) *Ochterus piliferus*, (e)–(g) *O. marginatus*). ((a) and (d)) CH1, CH2 are numerous and distributed dorsally (D) and ventrally (V) on the I and II segments of the labium. ((b) and (c)) PES are numerous and cover the III and IV segments of the labium in even rows, and between two rows of pegs there is a row of CUS. PAS2 are distributed on the labial tip. One pair of TRS2 is situated near the tip of the labium. (e) CH1, CH2 are densely distributed dorsally (D) on I and II segments of the labium. (f) PES are numerous and cover the III and IV segment of the labium in even rows, and between two rows of pegs there is a row of CUS. (g) PAS2 (no. 1–10) are on the labial tip; PIP is not visible. (h) COS (prioprereceptive sensilla) are situated on segment IV, dorsally, near the base of segment III.

**Figure 16:**
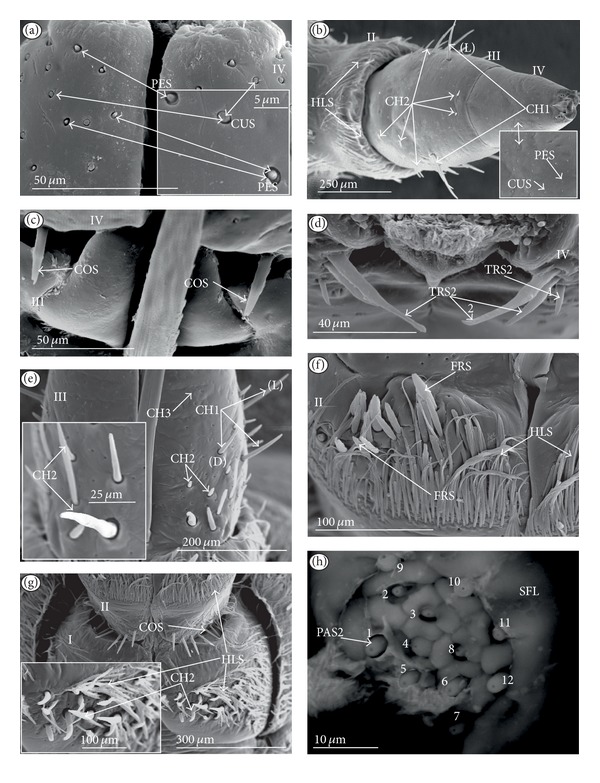
Types and sizes of the sensilla of the Gelastocoridae: Gelastocorinae (*Gelastocoris oculatus*). (a) On the dorsal surface PES are numerous and cover the IV segment of the labium in even rows, and between two rows of pegs there is a row of CUS. (b) CUS and PES are numerous and cover the ventral surface of the IV segment, a few CH1 are situated laterally (L) on the III segment, one pair of CH2 is placed ventrally (V), and a few CH2 are more situated more laterally on the III segment. (c) COS (prioprereceptive sensilla) are situated dorsally on the IV segment near the base of the III segment. (d) TRS2 (three) are situated ventrally near the tip of the labium. (e) CH1, CH2 are less numerous and situated dorsally (D) on the III segment of the labium, and CH3 are numerous and slightly visible. (f) Several FRS are present on the dorsal surface of the II segment, and HLS are very numerous and distributed in bands along the width of segment II. (g) CH2 and HLS are numerous and densely cover the dorsal surface on the labial segment I; COS are situated at the basal edge of the II segment (h) PAS2 (no. 1–13) are on the left SFL, and PIP in not visible.

**Figure 17:**
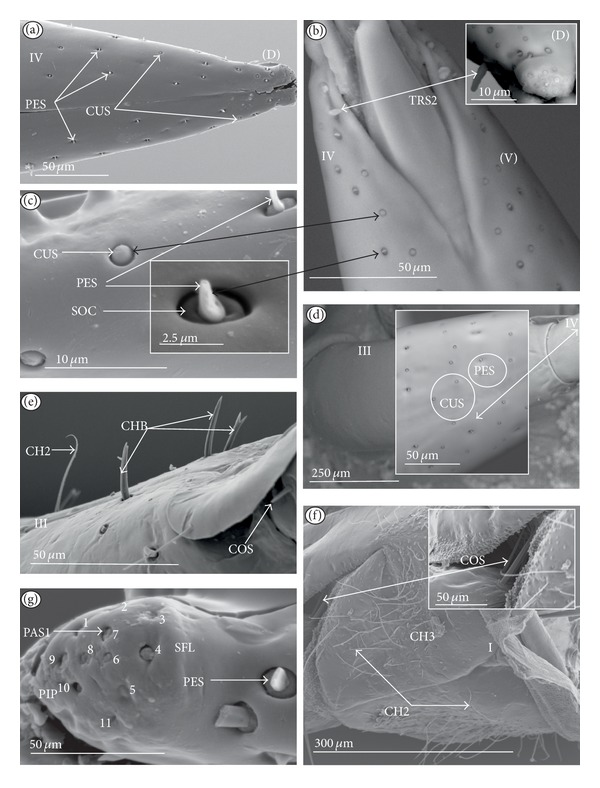
Types and sizes of the sensilla of the Gelastocoridae: Nerthrinae (*Nerthra nepaeformis*). (a) On the dorsal surface PES and CUS are numerous and evenly cover the IV segment of the labium. (b) TRS2 (one) is situated ventrally near the tip of the labium. (c) The shapes of CUS and PES, magnified. The socket is clearly visible. (d) PES and CUS are numerous and cover the ventral surface of the II and III segments. (e) CHB are unevenly distributed on the dorsal surface of the III segment, and COS (prioprereceptive sensilla) are situated on the dorsal surface of the IV segment near the base of the III segment. (f) CH2 are numerous and situated dorsally (D) on segment I of the labium, and COS are placed at the base of the edge of segment II. (g) PAS1 (no. 1–11) are distributed over SFL.

**Figure 18:**
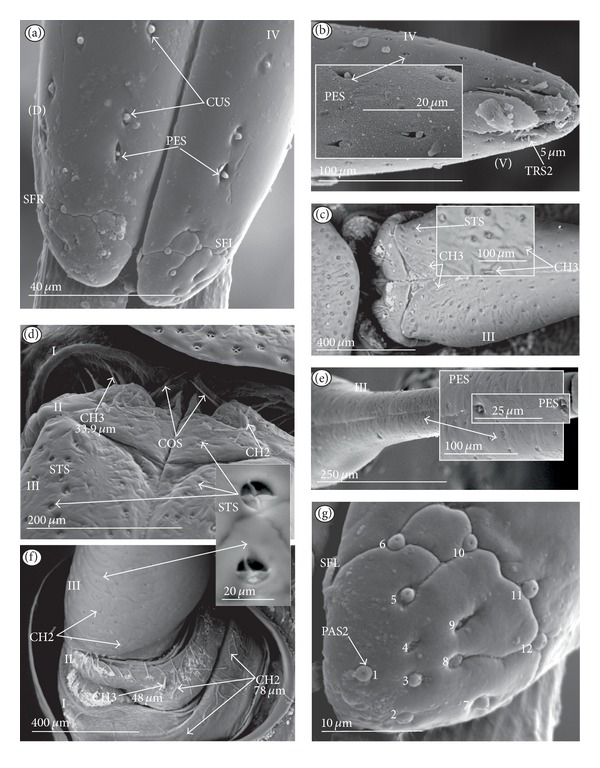
Types and sizes of the sensilla of the Aphelocheiridae (*Aphelocheirus aestivalis*). (a) PES and CUS are numerous and distributed unevenly in rows on the IV segment (dorsal view). (b) TRS2 (one) is situated ventrally near the tip of the labium; PES and CUS are distributed similarly as on the dorsal side. (c) STA and CH3 are in the proximal part of the III segment (dorsal view). (d) STS, CH2, CH3, and COS are situated on the II segment (dorsal view). (e) PES are unevenly distributed on the dorsal surface of the distal part of segment III. (f) CH2 are not numerous and are situated ventrally (V) on segments I and II of the labium. (g) PAS2 (no. 1–12) are distributed over SFL.

**Figure 19:**
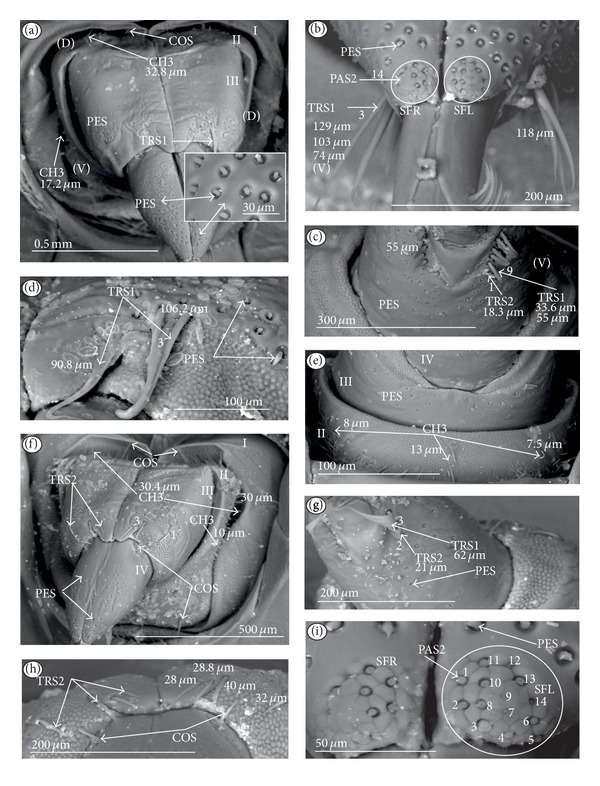
Types and sizes of the sensilla of the Naucoridae: Cheirochelinae ((a)–(e), *Cheirochela feana*, (f)–(i), *Gestroiella limnocoroides*). (a) PES are numerous and distributed evenly on the IV segment (dorsal view), PES are present only in the distal area of the III segment, CH3 are less numerous and distributed over the II segment (dorsally and ventrally), and COS are situated on segment II (dorsal view). (b) PAS2 (no. 1–14) are distributed over SFL and SFR; TRS1 (three) are visible only ventrally (V) (the first specimen). (c) TRS1 (nine) and TRS2 (one) are distributed ventrally (V) near the apex of segment IV (the second specimen), and PES are numerous. (d) TRS1 are distributed in two tufts (2 + 3) on the distal edge of the III segment, and several PES are visible near the TRS1. (e) CH3 are sparsely distributed over segment II; several PES are visible on the III segment (ventral view). (f) PES are numerous and distributed evenly over the IV segment, TRS2 (four) are distributed separately (3 + 1) on the distal edge of the III segment, several PES are visible near TRS1, CH3 are less numerous and distributed over the II segment, and COS are situated on segments IV and II (ventral view). (g) TRS1 (three) and TRS2 (two) are distributed ventrally (V) near the apex of segment IV. (h) TRS2 and COS magnified. (i) PAS2 (no. 1–14) are distributed over SFL and SFR.

**Figure 20:**
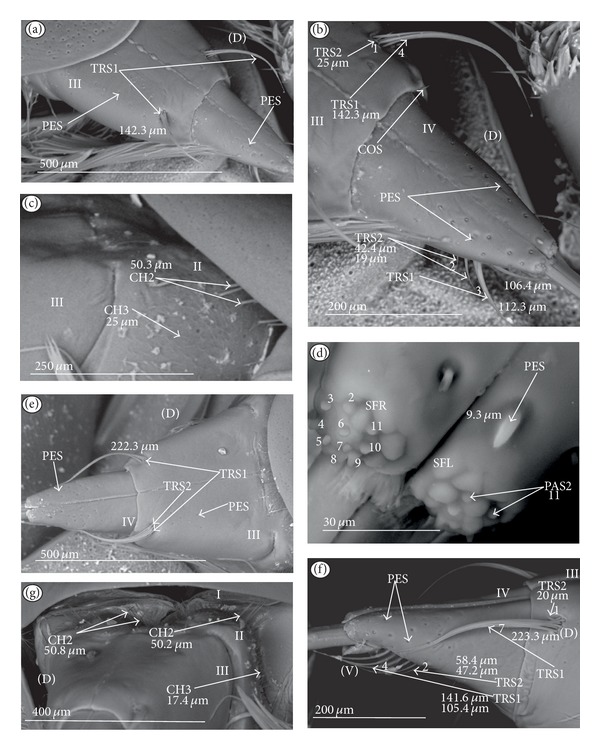
Types and sizes of the sensilla of the Naucoridae: Laccocorinae ((a)–(d), *Laccocoris hoogstraali*, (e)–(g), *Heleocoris humeralis*). (a) PES are numerous and distributed evenly on the IV segment (dorsal view). (b) TRS1 (three) and TRS2 (two) are distributed on the ventral (V) and dorsal (D) sides, and TRS1 (four) and TRS (two) are situated d in one tuft on the distal edge of the III segment. (c) CH3 densely cover the dorsal surface on the II segment; several CH2 are present. (d) PAS2 (no. 1–11) are distributed over SFL and SFR. (e) TRS1 and TRS2 are placed in one tuft on the distal edge of the III segment; PES are distributed evenly on the dorsal surface of the IV segment but are less numerous on the III segment. (f) PES are numerous and distributed evenly on the IV segment (lateral view); TRS2 (two) and TRS1 (four) are distributed ventrally (V) and dorsally (D); TRS1 (seven) and TRS2 (one) are distributed on the distal edge of the III segment. (g) CH3 densely cover the dorsal surface (D) of the II segment; only several CH2 are present.

*The Limnocorinae. Cryphocricinae, Naucorinae (Figures 7(f) and 7(g)).* On the labial segments (I, II, and III) the sensilla (CH2, CH3) are not numerous. On the IV segment the sensilla (MPS) are numerous (or not) and densely cover this segment while they are less numerous on the III segment. Ventrally, the sensilla are less numerous, except for the II segment, which in several species is frequently covered by CH2 and CH3 forming a dense row (Figures 7(g), 22(c), and 24(a)). Trichoid sensilla (several pairs of TRS1 and TRS2) are placed dorsally on the III segment as well as ventrally and subapically on the IV segment.

#### 3.8.4. Sensilla Are Not Numerous and Scattered Unevenly (Figures 7(h), 7(i), 7(j), 7(k), and 7(l))

*The Notonectidae, Pleidae, and Helotrephidae.* On the labial segments (I, II, and III) the sensilla (CH1, CH2, and CH3) are less numerous and scattered unevenly all over surface (they form a small group near the labial groove and around the distal edge of the III segment, dorsally). The IV segment is covered by numerous CH3. Only in the Notonectidae, on the ventral side of the I, II, and III segments the sensilla are numerous and form a dense layer. Trichoid sensilla (TRS1, TRS2) on the dorsal and ventral sides are placed subapically on the IV segment but in various numbers and sizes. Only in the Helotrephidae one pair of the TRS1 are situated medially on the dorsal side and a second pair of TRS1 is situated near the apex on the fourth labial segment (Figure 26(a)).

#### 3.8.5. Sensilla Are Very Numerous and Arranged in Transverse Bands or Scattered Unevenly on the Labial Surface (Figures 8(a), 8(b), and 8(c))

*The Corixidae (except for the Cymatiinae), Micronectidae, and Diaprepocoridae.* The sensilla (RBS1, RBS2) are distributed in several transverse bands (BD) on the triangular-shaped labium. In the Corixidae there are six transverse bands, in the Micronectidae there are four bands, while in the Diaprepocoridae there are two and a half bands. One band consists of two rows (r1, r2) of the semicircular grooves (GS) with one row of pore (p) (r3) and two rows (r4 and r5) of ribbon-like (RBS2, RBS1) sensilla. Peculiar characters have been observed in the Cymatiinae, where the sensilla are scattered over the smooth surface of the labium (RBS1, RBS2, and PES). Chaetica sensilla (CH1, CH2, and CH3) are distributed on the lateral and ventral sides. The trichoid sensilla (PES) have not been observed. 

### 3.9. Plan Distribution of the Prioprereceptive Sensilla (COS)

**Figure 21:**
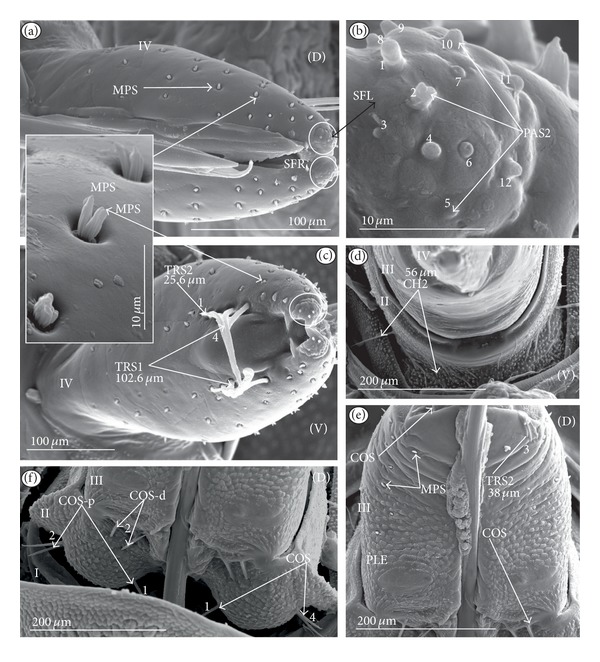
Types and sizes of the sensilla of the Naucoridae: Limnocorinae (*Limnocoris lutzi*). (a) MPS are numerous and distributed in rows over the IV segment (dorsal view). (b) PAS (no. 1–12) are located on the labial tip (SFL). (c) TRS1 (four) and TRS2 (one) are situated on the ventral side (V) in one row near the apex of the IV segment; MPS are also numerous ventrally and distributed in rows. (d) CH2 are less numerous and sparsely distributed over the ventral surface of the III and II segments. (e) TRS2 (three) are distributed separately on the distal edge of the III segment, MPS are less numerous and sparsely situated on the dorsal surface of the III segment, COS are situated on the IV segment, and on the surface of the III segment there are numerous, characteristic plates (generally unidentified structures, PLE-type). (f) Two (or four) COS-p are visible on the external distal edge of the II segment, and one is situated internally closer to the labial groove (Figure 21(f)). Two the COS-d are situated in the middle of the II segment on the proximal edge.

**Figure 22:**
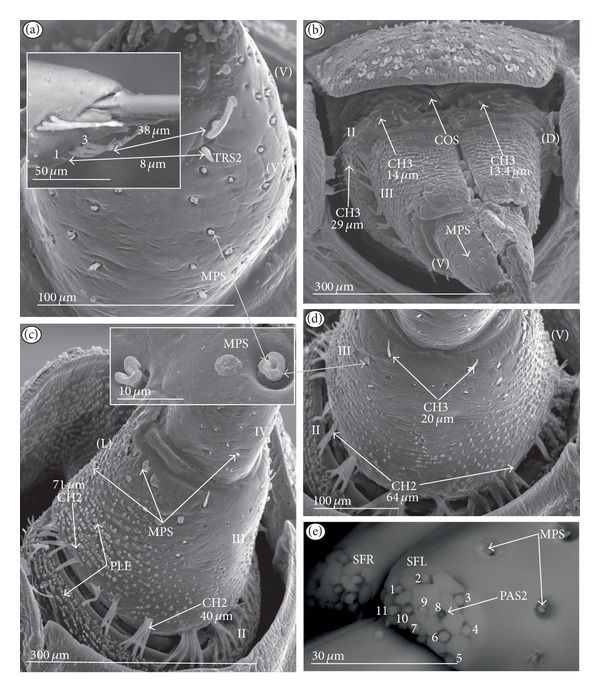
Types and sizes of the sensilla of the Naucoridae: Cryphocricinae (*Cryphocricos hungerfordi*). (a) MPS are numerous and spread evenly on the IV segment (ventral view); TRS2 (four) are distributed over the ventral side (V) in one row near the apex of the IV segment. (b) MPS are numerous and distributed evenly over the IV segment (dorsal view), CH3 are less numerous and situated on the dorsal surface of the II segment, and COS are situated on the II segment (dorsal view). (c) MPS are less numerous, and only a few are situated on the dorsal surface of the III segment; on this segment there are present numerous, unidentified plates (PLE); CH2 are situated around the edge of the II segment on the ventral side. (d) One pair of CH3 is situated ventrally near the distal edge of the III segment. (e) PAS2 (no. 1–11) are situated on the labial tip (SFL).

**Figure 23:**
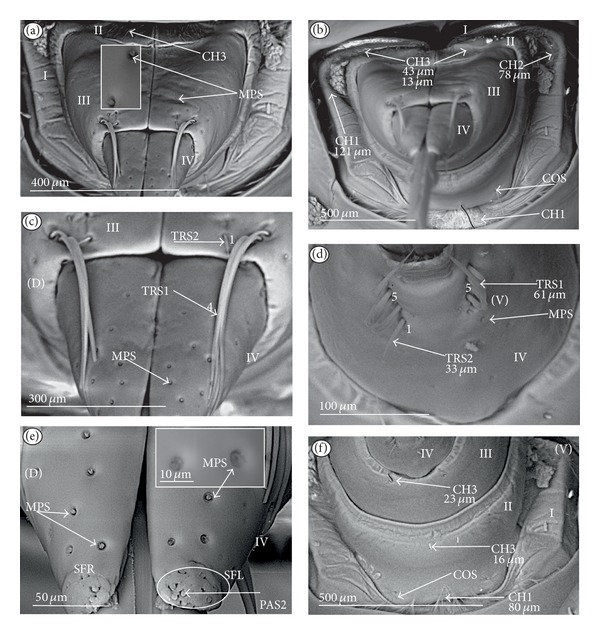
Types and sizes of the sensilla of the Naucoridae: Cryphocricinae (*Ambrysus occidentalis*). (a) MPS are less numerous and spread evenly over the III segment (dorsal view); several CH3 are present on the II segment (dorsal view). (b) CH2 and CH1 cover the dorsal and ventral surfaces of the I segment; COS are situated ventrally on the II segment. (c) TRS1 (four) and TRS2 (one) are situated on the dorsal side (D), in one tuft located distally on the III segment. (d) TRS1 (five) and TRS2 (one) are distributed over the ventral side (V) in one row near the apex of the labium. (e) MPS are less numerous and distributed evenly over the IV segment (dorsal view); PAS2 are distributed on the labial tip (SFR, SFL). (f) One pair of CH3 is situated ventrally near the distal edge of the III and II segments, CH1 are situated oil the edge of the I segment on the ventral side, and COS are located on the II segment on the ventral.

**Figure 24:**
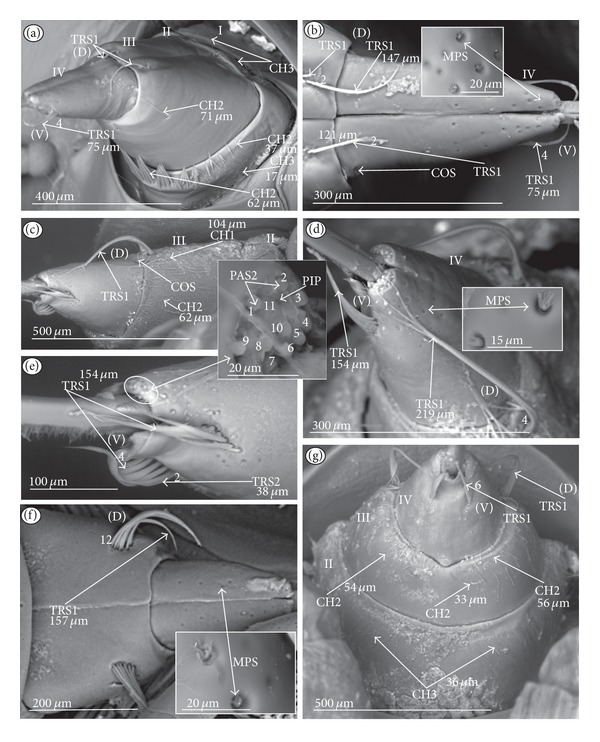
Types and sizes of the sensilla of the Naucoridae: Naucorinae ((a) and (b) *Naucoris maculatus*, (c)–(e) *Namtokocoris siamensis*, (f) and (g) *Neomacrocoris handlirschi*). (a) CH2 are numerous and form a collar around the distal edge of the II segment on the ventral side; individual CH2 can be seen on the III segment (lateral view); TRS1 and TRS2 are visible ventrally and dorsally. (b) TRS1 (four) are distributed over the ventral side (V) in one row near the apex of the labium; MPS are less numerous and spread evenly over the IV segment (dorsal view); TRS1 (two) are distributed over the dorsal side (D), in one tuft placed distally on the III segment; COS are situated dorsally on the IV segment. (c) CH2 and CH1 are densely spread over the ventral side of the III and II segments; COS are distributed dorsally over the IV segment. (d) TRS1 (four) are distributed over the dorsal side (D), in one tuft located distally on the III segment; MPS are numerous and spread evenly over the IV segment (dorsal view). (e) TRS1 (four) and TRS2 (two) are distributed over the ventral side (V) in one row near the apex of the labium; PAS2 (no. 1–10) and PIP (no. 11) are distributed on the labial tip. (f) TRS1 (twelve) arc distributed over the dorsal side (D), in one tuft placed distally on the III segment; MPS are less numerous and distributed evenly over the IV segment (dorsal view). (g) Several CH2 are visible on the ventral side of the II segment; less numerous CH3 are present on the II and III segments.

**Figure 25:**
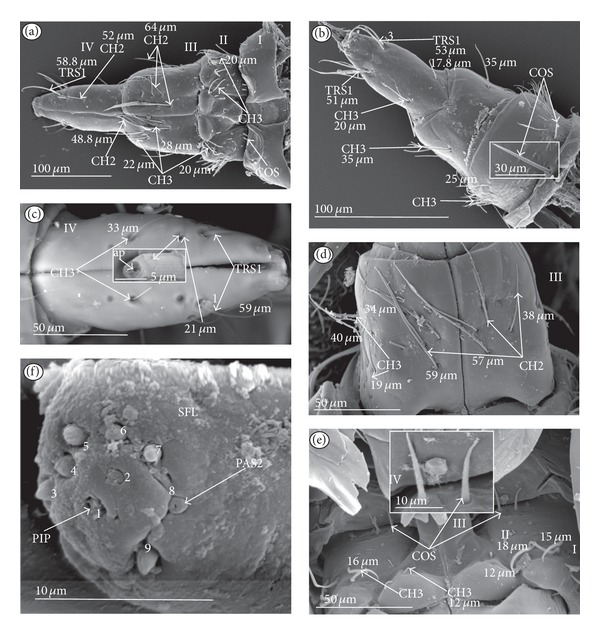
Types and sizes of the sensilla of the Pleidae (*Paraplea frontalis*). (a) CH2 and CH3 are less numerous and spread unevenly over all segment; TRS1 (one) is placed dorsally near the apex; one pair of COS is situated on the II segment. (b) TRS1 (three) are located ventrally near the apex; one pair of CH3 is situated near the proximal edge of the IV segment; one pair of CH3 is situated ventrally on the III segment; several CH3 are visible on the ventral side of the II segment, and some COS are located near the edge of the I segment; no sensila can be seen on segment I (ventral view). (c) TRS1 (one), TRS2 (one), and CH3 (two) are situated on the dorsal surface of the IV segment. (d) CH2 (several) are situated on the dorsal side and CH3 (several) are situated on the lateral side of the III segment. (e) Several CH3 are present on the III segment dorsally as well as one pair of COS. (f) PAS2 (no. 2–9) are placed in a circle on the labial tip, and PIP (no. 1) is situated centrally.

**Figure 26:**
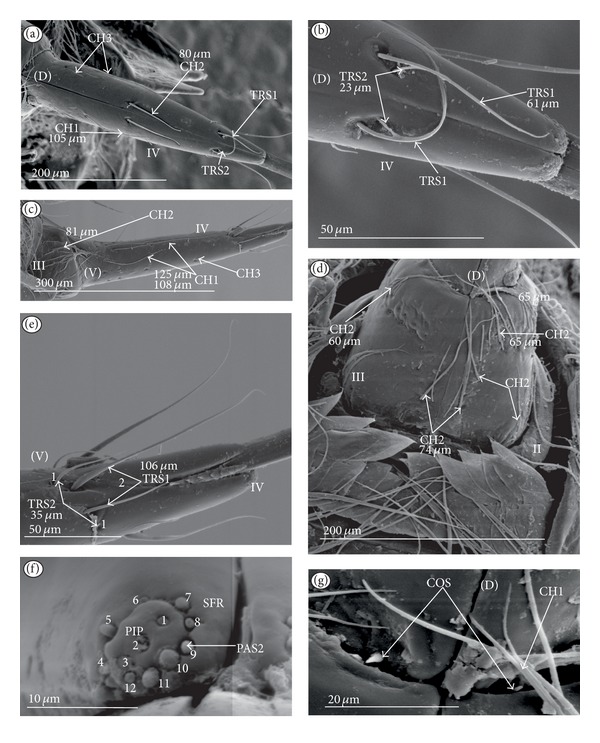
Types and sizes of the sensilla of the Helotrephidae (*Hydrotrephes visayasinensis*). (a) Several CH3, one CH2, and one CH1 are spread dorsally (D) oil the IV segment. (b) TRS1 (one) and TRS2 (one) are situated dorsally (D) near the apex. (c) Several CH3 and four CH1 are situated ventrally on the IV segment; several CH2 are situated on the III segment. (d) CH2 are placed near the labial groove and also near the distal edge of the III segment. (e) TRS1 (two) and TRS2 (two) are situated in two tufts, ventrally, near the apex of the labium. (f) Sensory field (SFR) with PAS2 (no. 1, 3–13) and PIP sensilla (no. 2). (g) COS are present on the dorsal surface of the IV segment, and several CH1 are present on the first segment.

**Figure 27:**
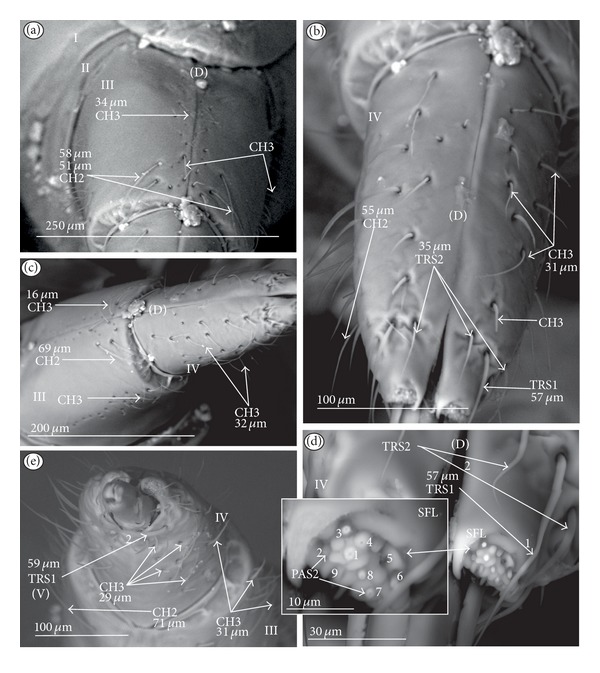
Types and sizes of the sensilla of the Notonectidae: Anisopinae (*Buenoa uhleri*). (a) Several CH3 and CH2 are present on the III segment (dorsal view). (b) Several CH3 and CH2 are present on the IV segment (dorsal view). (c) Several CH3 are scattered all over the surface of the IV segment (lateral view), two CH2 are situated near the distal edge of the III segment, and CH3 are situated in rows dorsally and laterally. (d) TRS1 (one) and TRS2 (two) are distributed over the dorsal side (D) near the apex of the labium; PAS2 (no. 1–9) are situated on the sensory field (left-side view; SFL). (e) Several CH3 are visible on the ventral side of the IV and III segments; TRS1 (two) are situated ventrally near the apex.

**Figure 28:**
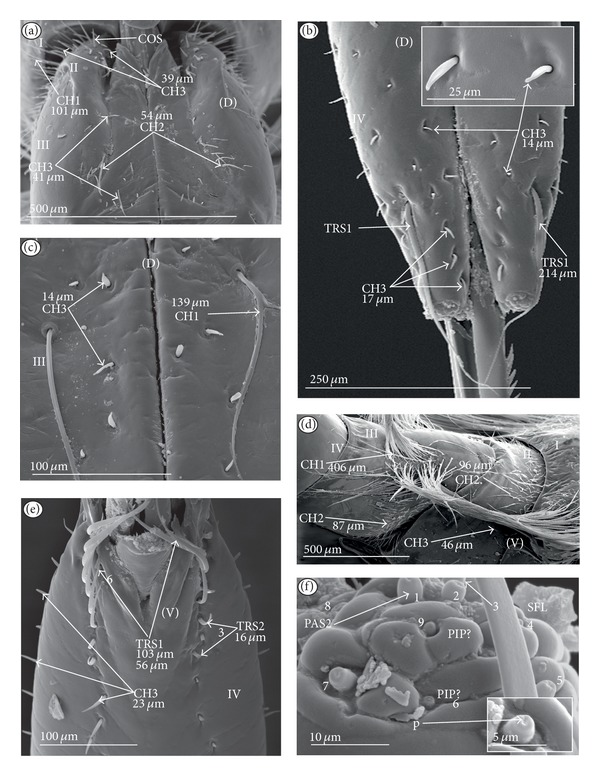
Types and sizes of the sensilla of the Notonectidae: Notonectinae (*Notonecta glauca*). (a) The arrangement of several CH2, CH3, and CH1 on the dorsal surface of the II segment; COS is situated near the edge of the I segment. (b) TRS1 (one pair) placed dorsally, near the apex; CH3 are numerous and distributed all over the surface of the IV segment (dorsal view). (c) CH1 and CH3 are situated in the distal part of the III segment. (d) CH1 are numerous and densely cover the ventral side of the II and I segments; CH2 are numerous and situated on the lateral side of the III segment; CH2 and CH3 are numerous and densely spread over the II and I segments (ventral view). (e) TRS1 (six) and TRS2 (three) are placed near the apex; CH3 are situated in two rows (ventral view, IV segment). (f) PAS2 (no. 1–7) and probably PIP (no. 6 or 9) are located on the sensory field (left-side view; SFL).

**Figure 29:**
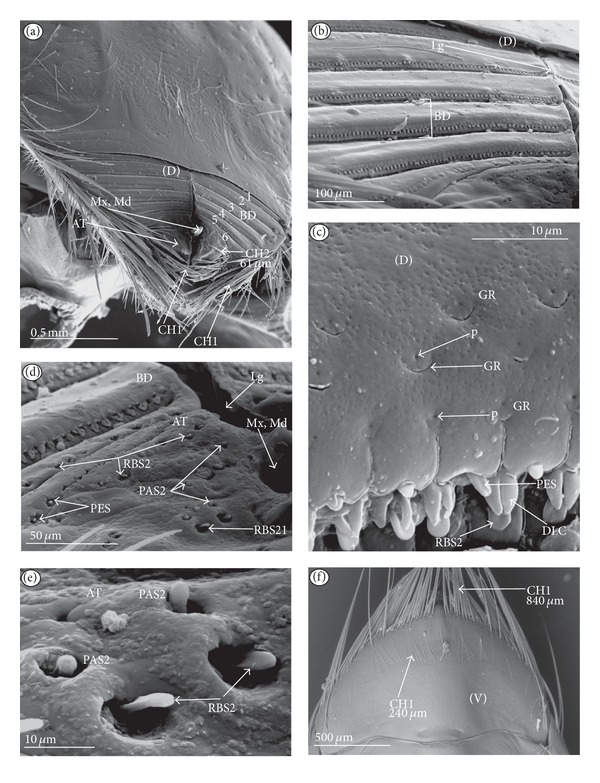
Types and sizes of the sensilla of the Corixidae: Corixinae (*Corixa dentipes*). (a) BD (no. 1–6) are spread evenly on the dorsal surface of the labium; several CH1 and CH2 are arranged on the lateral and ventral edges of the membranous labial lip (AT); CH1 located on the lateral edge of the labium below the apical tip. (b) BD magnified. (c) Three rows of porous sensilla (PLS) and RBS2 and RBS1 sensilla situated in two rows. (d) The apical tip (AT) with PES, PAS2, and RBS2 sensilla unevenly spread except for PES (they form a row). (e) PAS2 and RBS2 magnified. (f) Ventral view, CH1 are numerous and arranged on the lateral and frontal edges of the labium; shorter CH1 arranged in one row on the ventral side of the base of the labium.

**Figure 30:**
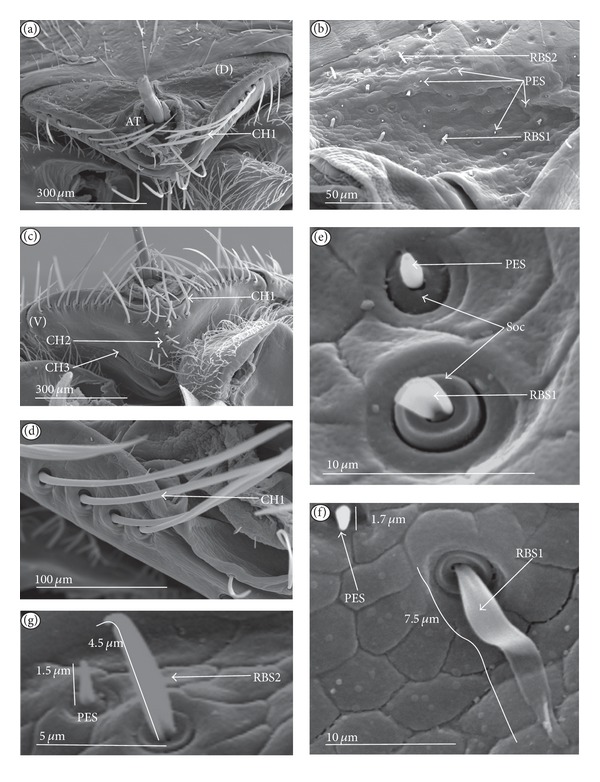
Types and size of the sensilla of the Corixidae: Cymatiinae (*Cymatia coleoptrata*). (a) Several of the CH1 are placed on the lateral edge of the labium and below the apex; several PES and RBS2 are visible on AT. (b) RBS1, RBS2, and PES are spread unevenly on the dorsal surface of the labium. (c) CH1, CH2, and CH3 are less numerous and situated ventrally. (d) CH1 magnified (lateral view). (e) PES and RBS1 magnified, Soc: socket. (f) RBS1, magnified. (g) The sizes of PES and RBS2.

**Figure 31:**
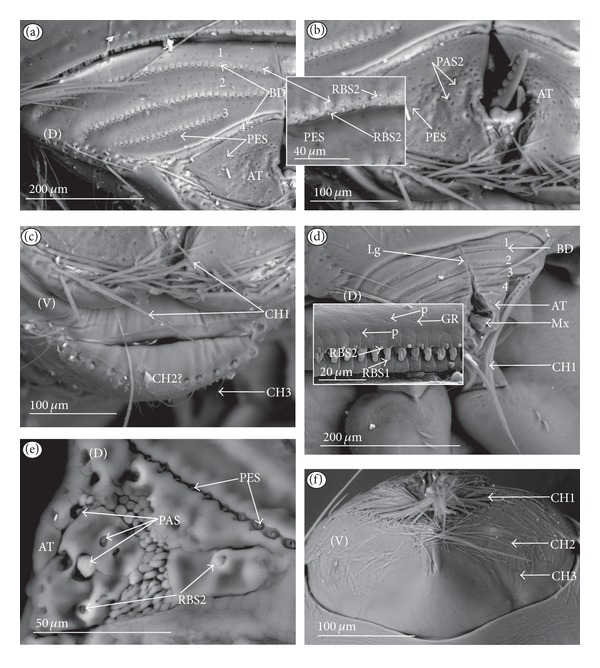
Types and sizes of the sensilla of the Diaprepocoridae and Micronectidae ((a)–(c) *Diaprepocoris zealandiae*, (d)–(f) *Micronecta quadristrigata*). (a) BD (no. 1−2.5) are spread evenly on the dorsal surface. (b) AT in magnification; PES or PAS2 are numerous and unevenly cover the tip. (c) Several of the CH1 are arranged on the lateral edge of the membranous labial tip (AT); the CH1 are placed on the lateral edge of the labium; the CH2 and CH3 are placed below the apex, on the ventral side. (d) BD (no. 1–4) are spread evenly on the dorsal surface; the CH1 are placed on the lateral edge of the labium. (e) BD in magnification, PES, RBS2, and PAS on the apex (AT). (f) The CH1 are numerous and situated below the apex (ventral view), the CH2 are situated in a short row below the CH1, and the CH3 are not numerous and placed below the CH2.

The one pair of prioprereceptive sensilla (COS) is restricted to the dorsal side of the second and fourth segments of the labium in most nepomorphan species, except for the Corixoidea (these sensilla have not been found). In the proximal part of the second and fourth segment there is usually one pair of the prioprerecptive sensilla located dorsally near the rear edge of the segment. However, in the Nerthrinae three pairs are visible on the second segment. In *Limnocoris lutzi* COS are present in three locations. Two (or four) of the COS-p are on the external distal edge of the II segment, and one is placed internally closer to the labial groove (Figure 21(f)). Two COS-d sensilla are situated in the middle of the II segment on the proximal edge. The prioprereceptive sensilla on the ventral side of the labium between I and II segment are visible in the Helotrephidae.

## 4. Discussion

 The present study carriers out a comparative analysis of the labial sensilla in water bugs, the Nepomorpha. The labium is equipped with a set of sensilla, including a large group of different types of the mechanosensilla and a smaller group of the chemosensilla.

### 4.1. The Sets and Distribution Types of the Labial Sensilla in the Nepomorpha

The total number of chemosensilla at the tip of the labium and the plan of their distribution allows the recognition of five main groups in the Nepomorpha and supplies evidence for the relationships between these groups. The first group consists of belostomatids, nepids, and the *Nerthra*. However, the *Nerthra* systematically belongs to the Gelstocoridae and it is rather unexpected that it shows similarity of the labial tip sensilla to those found in belostomatids and nepids. The second group includes the ochterids and aphelocheirids. The third group includes the representatives of gelastocorids, naucorids, and notonectids whereas in the fourth group there are pleids and helotrephids. The fifth group is totally different from the above-mentioned types. In the Corixoidea, the triangular labial tip has several PAS2 and PES sensilla, unevenly spread. In this hierarchy of the discussed taxa there can be seen general conformation of families or groups of families to the pattern of close relationships, which have been indicated in previous studies conducted by other researchers [30–32].

The trichoid sensilla are usually present in most taxa but in different numbers (Table 2). They are located on the dorsal and ventral surfaces on the fourth segment of the labium (near the apex) in the families of Belostomatidae, Pleidae, Helotrephidae, and Notonectidae. In addition, in the Nepidae trichoid sensilla are present also on the lateral sides near the apex. In three other families, namely, the Ochteridae, Gelastocoridae, and Aphelocheiridae these sensilla are observed only on the ventral side of the fourth segment. Furthermore, other differences are visible also in the Naucoridae, where the trichoid sensilla are situated on the third segment of the labium in the dorsal position, but ventrally they are situated on the fourth segment. In the Corixoidea the trichoid sensilla have not been recognized. Probably they are not visible being hidden in the dense layer of the chaetica sensilla.

The most conspicuous and common in the water bugs (Belostomatidae, Nepidae, Naucoridae, Pleidae, Helotrephidae, and Notonectidae) are chaetica sensilla covering the first, second, and third labial segment. In four cases chaetica sensilla are also present on the fourth segment of the labium for example, in the Nepidae, Pleidae, Helotrephidae, and Notonectidae. Moreover, the chaetica sensilla are restricted only to the first and second labial segments in the Ochteridae and Aphelocheiridae. Generally, the chaetica sensilla (CH1, CH2, and CH3) are distinctly differentiated on the dorsal and ventral surfaces of the labium and placed in small tufts in narrow rows and frequently are scattered unevenly on both surfaces. The major difference between the chaetica sensilla in the Belostomatidae and the chaetica sensilla in the remaining families is that in the case of chaetica sensilla in all examined species of the Belostomatidae there are distinct areas where they are present (the sensilla are grouped on the dorsal side of the I and II segment or arranged along the groove of the labium on the III segment) and these sensilla are numerous in contrast to those scattered all over the surface of the labial segments in some remaining taxa, except for the Pleidae, Helotrephidae, and Notonectidae. In the above-mentioned taxa the system of distribution of the sensilla is similar to the Belostomatidae, but the sensilla are less numerous. The evidently different pattern of distribution of the chaetica sensilla (densely spread over the lateral side of the labium and around the membranous labial tip) can be seen in the Corixoidea.

However, several shapes of mechanosensilla different from the chaetica sensilla have been noted in representatives of different subfamilies (Table 2). Among nepomorphan taxa in the Nepidae the squamiform sensilla and paddle-like sensilla (PDS) are the most abundant over the entire labial surface, and they form a very dense coat. Moreover, this taxon represents another peculiar type of sensilla on the II and III segment such as trichobothrium sensilla (TBS) and basiconicum sensilla (BAS) as well as the clubbed-like sensilla (CBS) positioned on the IV segment. Moreover, rich and diverse sensory equipment has been revealed in the Gelastocoridae, with three peculiar types of sensilla such as the freniale-like (HLS), finger-like (FRS), and the chaetic sensilla with a bisected tip (CHB). Another example of the presence of a unique sensillum was the star-like sensillum on the II and III segments in the Aphelocheiridae. In addition, in most parts of the nepomorphan taxa (except for the Nepidae, Limnocorinae, Cryphocricinae, Naucorinae, and corixoids species) on the IV labial segment the cuppola-like sensilla and peg sensilla are dominant. The multilobe sensilla (MPS) are typical only in the Limnocorinae, Cryphocricinae, and Naucorinae, whereas ribbon-like senilla (RBS1, RBS2) have been documented only in the corixoids. The term “ribbon-like sensilla” in this paper has been adopted from that used by Brożek [24]. Earlier works [10, 13, 14] used the term “peg sensilla” for the sensilla placed in transverse bands in the corixids, however, the latest observations of the shapes of these sensilla have lead to a conclusion that their shapes distinctly deviate from the shape of a peg.

The prioprereceptive sensilla (COS) are visible in most nepomorphan species except for the Corixoidea. Their position on the dorsal side of the fourth segment of the labium is generally stable in most representatives of the Nepomorpha. In the proximal part of the third segment there is usually one pair of the prioprerective sensilla, located dorsally near the rear edge of the segment. However, only in the Nerthrinae the three pairs on the third segment are visible, and several (i.e., five) of those sensilla in slightly different positions can be observed in *Limnocoris lutzi*.

### 4.2. The Ground Plan of Nepomorphan Labial Sensilla

The Belostomatidae and Nepidae have been coded as taxa possessing basal characters in the ground plan of the morphological approach used by Rieger [33], Popov [2], and Mahner [32] as well as the approach developed by Hebsgaard et al. [30] combining morphological and genetic data. Thus, some of the characters in these taxa may theoretically represent a plesiomorphic condition.

In the absence of data on the labial sensilla from the outgroup (the Enicocephalomorpha have been proposed as a basal outgroup by Štys [34, 35], Schuh and Slater [9], after Wheeler et al. [36] and in this study the nepomorphan ground plan of the labial sensilla is modeled on the sensory equipments of the Belostomatidae and Nepidae represented by the following six sensilla structures:

1. apical sensory field with 10–13 pairs of papilla-like sensilla (PSA1);
2. apical sensory field with the peg-in-pit (PIP) sensilla placed rather laterally on the sensory field;
3. numerous trichoid sensilla placed on the IV segment on the dorsal, lateral and ventral sides;
4. chaetica sensilla (CH1, CH2, and CH3) present and placed in groups or rows distributed along the labium near the labial groove on the dorsal side and also several chaetica sensilla unevenly scattered on the surface of that segment;
5. cupola (CUS) and peg (PES) numerous and evenly scattered over the labium;
6. prioprerective sensilla (COS): one pair positioned on the dorsal side, on the fourth segment of the labium.

In all analyzed species (fifty five), it has been discovered that the labial tip sensilla are very similar and a common pattern can be established for species which belong to the same family or group of families that are closely related. Consequently, for nepomorphan taxa the five patterns of the distribution of the labial tip sensilla can be presented (above). In comparison with the other heteropteran infraorders it has been observed that the morphology and distribution of the labial tip sensilla in the Nepomorpha are distinctly varied with respect to what has been reported in the Gerromorpha (Brożek and Zettel, in prep), Pentatomomorpha [19, 21], and Cimicomorpha: Reduviidae [15, 25]. In terms of diversity and abundance of the sensory system on the labial tip, the Nepomorpha come second to the Gerromorpha. In gerromorphan bugs a richer and more diverse sensory equipment (several morphological types of sensilla) has been detected [Brożek and Zettel, in prep]. An undifferentiated sensory system occurs in the Pentatomomorpha (e.g., *Dysdercus fulvoniger* and *D. koenigii*, *D. fasciatus, D. intermedius, Lygus lineolaris, and Neomegalotomus parvus*), where the labium tip usually represents one morphological type of basiconic sensilla functioning as the contact-chemoreceptive sensilla (several pairs), basiconic gustative sensilla (several pairs), and basiconic olfactive sensilla (one or two pairs) [17, 19, 21, 22, 37]. In some of the Reduviidae one type of peg and peg-in-pit sensilla have been noticed [15, 25, 38]. Furthermore, two types of mechanosensilla (small hairs and long bristles) have been identified on the tip of the labium by Bernard [39].

The chaetica sensilla on labial segments in the Nepomorpha (i.e., Belostomatidae, Corixidae, some of Naucoridae, Pleidae, Helotrephidae, and Notonectidae) closely resemble these in the Reduviidae (Peiratinae and Triatominae) [15, 25] as well as in other hemipteran groups, for example, the Fulgoromorpha and Cicadomorpha [40–42]. Other nepomorphan taxa (Nepidae, Gelastocoridae, Aphelocheiridae, and some of the Naucoridae) have modified base and apical “hairs”, which represent various forms of mechanosensilla. Examples for various types of hairs are found in many insects [8], where the tip of the hairs is enlarged, disk-shaped, or divided. Probably the large variety of labial types of mechanosensilla of the Nepomorpha can indicate that the sensilla evolved several times within that group and they can be interesting as a phylogenetic signal. The inspection of chaetica sensilla in the nepomorphans suggests that the number and type of their distribution are quite diverse among species. Similar data have been obtained during the observation of chaetica sensilla in several species of the Triatominae [1, 25, 38, 43]. The more established pattern of the distribution of chaetica sensilla on the apical segment of the labium has been documented in the Peiratinae: 19 species were revealed to represent three patterns of distribution [15]. According to the present study, the type of the arrangement of chaetica sensilla with the other type of mechanosensilla recognized on the subfamilies level is stable. Consequently, five possible types for particular taxa have been reported (see above).

Previous examination of the species representing the Peiratinae and Triatominae [15, 25, 38] have shown a lower number of the trichoid sensilla (one or two pairs in all species) on the dorsal and ventral sides than is observed in most nepomorphan taxa. Presently, the study corroborates and broadens the range of distribution of the trichoid sensilla in some nepomorphan species previously described by Brożek [24]. The trichoid sensilla are characteristic with respect to their number and position on the labium in different species in particular families (Table 2). Judging from the way the trichoid sensilla are oriented and bent in the basal and apical positions, the hairs on the ventral and dorsal side are the first to touch the victim in most nepomorphan species. The trichoid sensilla and long chaetica sensilla (type 1) situated on the labium in the Reduviidae work in a similar way [25]. However, in four nepomorphan taxa (Ochteridae, Gelastocoridae, Aphelocheiridae, and Naucoridae) the identification of victims is performed only by the ventral trichoid sensilla. In this case, it can be explained that different species show different responsiveness to host movements, which might be related to the types and numbers of sensilla present. It is known that there are variations in feeding strategies in hemipteran insects, and these strategies are strongly correlated with taxonomic position and therefore have evolved in distinct patterns [1, 25, 44]. In connection with this fact it is logical that the sensory systems that mediate feeding have also evolved in patterns. The sensilla used to detect external plants or animals, responsible for the cues to host orientation and host acceptance (antennal and labial sensilla), are highly variable among species of the different taxa [40, 41]. Similar conclusions have been reached for other groups of insects [4]. 

### 4.3. Evaluation of the Significance of the Characters of Labial Sensilla in the Systematic Groups

The cladistic analysis of morphological and molecular data on the Nepomorpha given by Hebsgaard et al. [30] and the analysis of their mitochondrial genome presented by Hua et al. [31] provide very different relationships proposals of the families.

Providing new characters can significantly influence the future cladistic analysis of the Nepomorpha. A wide variety of labial types of sensilla, especially mechanosensilla, are observed among the Nepomorpha, and their types of distribution can indicate that sensilla have evolved several times within that group and they can bear high phylogenetic signal together with the other characters. However, the current study, limited to the one set of features, is insufficient to form a new phylogenetic hypothesis. The presented data on sensory organs characters do not allow to support or reject the proposals of Hebsgaard et al. [30] and Hua et al. [31] and also of older studies [2, 32, 33]. Obviously, several significant indications regarding the relationship of certain families of the Nepomorpha can be pointed out based on the current characters (apomorphies).

#### 4.3.1. Mechanosensilla of Specialized Shapes

The characters (except for characters in the ground plan) distinguished in the individual taxa are therefore to be regarded as advanced. Consequently, the presence of the modified shapes of the sensilla and their various distribution types should be interpreted as the apomorphic character states. It is probable that the fourteen specialized sensilla (presented below) should be regarded as ones that have evolved from plesiomorphous peg-like sensilla or/and cupola sensilla.

 The recognition of four types of sensilla (SQS, TBS, BAS, and CBS) in the Nepinae and the paddle-like sensillum (PDS) in the Ranatrinae has been estimated as autapomorphies of these taxa. The squamiform sensillum (SQS), in the Nepinae, is probably homologous to the paddle-like sensillum in the Ranatrinae. Both types (SQS and PDS) are evidently similar morphologically; they are sunken into the surface and clearly numerous. In addition, they densely cover the whole surface of the labium. The clubbed-like sensillum (CBS) slightly protrudes from the surface and has probably evolved from the peg sensilla of the Belostomatidae. The basiconic and trichobothrium sensilla are frequently observed in other insects on various body parts; they might represent a homoplasy. The autapomorphical characters of these sensilla (SQS, CBS, and PDS) could well serve for the confirmation of the monophyly of both subfamilies. In an earlier study the monophyly of these taxa was not questioned [30]. 

Generally, the Corixoidea is regarded as a taxon with several advanced characters [2, 30, 45], and presently a trend has been demonstrated towards changes in the type and distribution of the sensilla on the labium in comparison to other nepomorphan taxa.

The modified sensilla types (ribbon-like sensilla) are situated either in transverse bands in the Corixinae, Diaprepocoridae, and Micronectidae or without bands in the Cymatiinae. Presumably, in the first case it is a synapomorphy for the Corixidae (except the Cymatiinae), Diaprepocoridae and Micronectidae, and in the second case it is an autapomorphy for the Cymatiinae. The number of bands can be a crucial factor among the taxa and supply autapomorphical characters for these families. Currently, in each of the six species of the Corixidae, six bands have been counted, and in the Micronectidae (*Micronecta quadristrigata*) four bands have been found while in the Diaprepocoridae (*Diaprepocoris zealandiae*) two and a half bands have been observed. As for other members of the Corixidae, Lo and Acton [14] pointed out six bands in *Cenocorixa bifida* while in the same species Jarial [13] pointed out seven bands.

The presence of chaetica sensilla on the lateral side of the labium represents the synapomorphic character for these taxa. It is probable that these sensilla (i.e., ribbon-like sensilla) evolved from a more plesiomorphous peg-like sensillum or cupola-like sensilla of belostomatids. On the other hand, the sensilla of corixids are more similar to the paddle-like sensilla of the Ranatrinae, and a transformation of the characters directed from nepids to corixids could be indicated. A strong modification of the shape of the labium, diet type [2, 11, 45], modification of the food pump structures [33], and presently modification of the shape and distribution pattern of the sensilla of corixids rather lead to a conclusion that this group belongs to the advanced evolutionary line. These assumptions contradict those of Hua et al. [31], who interpreted the Corixidae as the basal taxon.

The Ochteridae have retained the plesiomorphic set of characters of chaetica sensilla but they have a clearly different distributional pattern of the cupola and peg sensilla: they are arranged in six regular rows along the last segment of the labium, which can be treated as their apomorphic feature with respect to the basal morphological plane of the sensilla. The cupola and peg sensilla in the Gelastocoridae have the same pattern as in the Ochteridae, which indicates a possible synapomorphy. An interesting process of differentiation of the mechanosensilla can be seen in the Gelastocoridae. In the Gelastocorinae the two new types of sensilla (FRS, HLS) could be considered as autapomorphies, while in the Nerthrinae the chaetic sensillum with a bisected tip (CHB) has the autapomorphical character. Apart from that, the peculiar shape of the star-like sensillum (divided into four plates), which has evolved only in the Aphelocheiridae, is regarded as an autapomorphy. Further changes regarding the shape of mechanosensilla have to be postulated within subfamilies of the Naucoridae. In the Cryphocricinae, Limnocorinae, and Naucorinae, the multilobe sensilla on the IV segment are present and have been identified as synapomorphy for them, in contrast to the Cheirochelinae and Laccocorinae with the peg sensilla on the IV segment (plesiomorphic condition). The relationship of these taxa is unclear in this arrangement [26], and further discussion in this area appears premature. In the opinion of Hebsgaard et al. [30], the Naucoridae is a monophyletic family. This hypothesis probably cannot be supported by synapomorphic characters found for the two groups: the first comprising the Cheirochelinae and Laccocorinae and the second comprising the remaining subfamilies: the Cryphocricinae, Limnocorinae, and Naucorinae.

 The chaetic sensillum with a bisected tip (CHB) in the Nerthrinae possibly provides support to the star-like sensilla in the Aphelocheiridae, with the tip deeply divided into four narrow lobes, which in turn may be a prerequisite for the further transformation into the multilobe-like sensilla of the Cryphocricinae, Limnocorinae, and Naucorinae.

The peg sensilla are common in a few taxa of the Nepomorpha, such as the Belostomatidae, Ochteridae, and Gelastocoridae, and in the Corixoidea. Assuming that the peg sensilla and cupola sensilla are present on the ground plan of nepomorphans in basal families, the loss of these structures which can be observed within the Pleidae, Helotrephidae, and Notonectidae points out synapomorphies for these taxa. 

#### 4.3.2. The Chaetica Sensilla

Chaetica sensilla (CH1, CH2, and CH3) on the I, II, and III segment, have been documented in most taxa while the cupola and peg sensilla or other peculiar sensilla have been distinguished usually on the IV segment of the labium and rarely on the III segment. The presence of the chaetica sensilla has been pointed out on the IV segment only in the Nepinae, Notonectidae, Helotrephidae and Pleidae as a similar structure of the previous segments. Nevertheless, chaetica sensilla without other types of the mechanosensilla (except for prioprereceptive sensilla) are present only in the Notonectidae, Helotrephidae, and Pleidae, and these structures might represent an evolutionary novelty. The systematic distribution only of the chaetica sensilla on the IV segment only in the Notonectidae and Pleoidea (Pleidae + Heloterphidae) points to the origins of this structure in these taxa. Probably, this character can be estimated as synapomorphy for this group of families. In fact, chaetica sensilla have apparently evolved at least twice within the Nepomorpha, on the I, II, and III segment in several groups (Belostomatidae, Nepidae, Corixidae, Diaprepocoridae, Micronectidae, Ochteridae, Gelastocoridae, Aphelocheiridae, and Naucoridae) and on the IV segment in representatives of the Pleidae, Helotrephidae, and Notonectidae. Previous studies strongly supported the view of separated superfamilies Notonectoidea (including one family Notonectidae) and Pleoidea (including the Pleidae and Heloterphidae), recognized by China [45], Popov [2], Rieger [33], and Hebsgaard et al. [30]. The presently reported new synapomorphy of these three taxa rather supports the existence of the “superfamily” Notonectoidea (Notonectidae + Pleoidea (Pleidae + Helotrephidae) which is similar to what was proposed by Mahner [32].

#### 4.3.3. Trichoid Sensilla

This study documents the existence of four distinctly different types of attachment of trichoid sensilla (TRS) in the nepomorphan labium. They are as follows: the first, TRS on the fourth segment (in dorsal and ventral sides as well as on the lateral side), is restricted to the Nepidae (autapomorphy); the second in dorsal and ventral sides is restricted in the four families (Belostomatidae, Notonectidae, Pleidae, and Helotrephidae) and represents a plesiomorphic character; the third, TRS on the fourth segment only in the ventral position (Ochteridae, Gelastocoridae, and Aphelocheiridae) represents synapomorphies for these taxa; the fourth, TRS on the third segment in the dorsal position, is a novelty and is an autapomorphy for the Naucoridae; similarly, the absence of the TRS in the Corixoidea could be an autapomorphy for this taxon. 

 A general separation of the families in the light of the types of distribution of the trichoid sensilla is similar to their systematic status. The analysis of the trichoid sensilla in particular families or subfamilies has shown that the number of these sensilla is a differentiating factor for these taxa. In the Belostomatidae, numerous trichoid sensilla have been observed (3–8 dorsal, 11-12 ventral pairs) which is similar to some of the Naucoridae (4–12 dorsal and 4–12 ventral pairs), and there is a tendency for the reduction in their number in the remaining groups, for example, in the Nepidae (4–6 dorsal and 4–6 ventral pairs and lateral 4 pairs), Notonectidae (2–9 dorsal, 1–5 ventral pairs), and Pleidae and Helotrephidae (1–3 dorsal and 1–3 ventral pairs), as well as in the Ochteridae, Gelastocoridae and Aphelocheiridae (2 ventral pairs).

 A significant loss of the trichoid sensilla in the Ochteridae, Gelastocoridae, and Aphelocheiridae could, therefore, fit in with their common evolutionary trend. The Ochteridae and Gelastocoridae—neither of which has trichoidea sensilla at the dorsal side—have long been thought to be closely related to each other based on other characters [2, 30–33, 45]. In turn, the Aphelocheiridae, albeit having the general appearance of many naucorids, they differ in some characters, particularly, the relative length of the labium. On the other hand, similar lengths of the labium are characteristic for the Ochteridae and Aphelocheiridae [9, 46], and thus it seems that labial sensilla might have evolved simultaneously with the labium. Also trichoid sensilla at the third segment in the Naucoridae can have evolutionary significance; they have probably evolved independently.

#### 4.3.4. Papillae and Peg-in-Pit Sensilla

The papillae and peg-in-pit sensilla can be found together only on the labial tip. The papillae sensilla (PAS1) are situated on the smooth tip in the Belostomatidae, Nepidae, and Nerthrinae (Gelastocoridae), and sensilla (PAS2) are situated at the folded tip in the remaining families. The labial tip of nepomorphan families shows a fairly homogeneous distribution of PAS1 and PAS2 sensilla as well as morphological similarity and identical function. At the same time, the peg-in-pit (PIP) sensillum can be observed in lateral position in the Belostomatidae and Nepidae or in the central position on the labial tip in most of the remaining taxa. 

Another aspect of this sensillum (PIP) is the possibility of its presence in all taxa. On the basis of the present documentation this type of sensillum does not occur in the ochterids, gelastocorids, and aphelocheirids. It is possible that it could be hidden in the folded labial tip in these groups and therefore has not been identified in them. Due to the presence of this type of sensillum in most representatives of nepomorphans it can be regarded as putative symplesiomorphy for them.

 As far as other heteropterans are concerned, the papillae sensilla do not occur in the Gerromorpha [Brożek and Zettel, in prep], Pentatomomorpha [16–19], and the Cimicomorpha [15, 25], but very similar short peg-like sensilla and basiconic-like sensilla have been reported to exist on their labial tips. Furthermore, in the above-mentioned groups the peg-in-pit sensillum has been observed. Assuming the conservative approach to these observations I consider that the papillae sensilla (PAS1 and PAS2) are homologous in all nepomorphans. According to the currently accepted phylogeny of the group [30], the PAS1 papillae sensilla represent, therefore, the plesiomorphic state (Belostomatidae + Nepidae), from which at least new apomorphically-shaped PAS2 sensilla have evolved. The presence of the PAS1 can be considered as a possible symplesiomorphy for the clade Belostomatidae + Nepidae and for the Nerthrinae, but the PSA2 are a synapomorphy for the rest of the Nepomorpha. Assuming that the Gerromorpha is the sister group of the Nepomorpha [36] and peg-in-pit sensilla (PIP) are homologous in both infrorders, as well as in the remaining advanced infraorders of the Cimicomorpha (e.g., in the Peiratinae and Triatominae), the presence of the PIP is probably a symplesiomorphy for the Neoheteroptera and Panheteroptera. However, more morphological studies of the heteropteran labial sensilla in all these groups are needed before positive conclusions can be reached.

### 4.4. The Labial Sensilla and Their Function

#### 4.4.1. Mechanosensilla

The sensilla described in the present study are referred to as ribbon-like sensilla in the corixids while in the works by Benwitz [10], Lo and Acton [14], and Jarial [13] are referred to as peg sensilla. The ribbon-like sensilla are classified as mechanosensilla (they lack pores and are sunken in the socket) and evidence of this morphological similarity has also been found in the case of peg sensilla [14]. The ultrastructure of labial sensilla (peg) in the *Cenocorixa bifida* does not allow, according to Lo and Acton [14], the identification of their exact function. The modified ciliary structures observed in the dendrites are known to occur in many different receptors (photoreceptors, mechanoreceptors, and chemoreceptors). In several, out of over 2000, sensilla the cuticle is permeable to a solution of crystal violet, which makes it possible that these sensilla have a chemoreceptive function. In the case of most peg sensilla, the function is not known. Another possible function of the transverse bands of sensilla on the labium might be associated with osmotic and ionic regulations [14]. According to Jarial [13], the semicircular groove with the pores and numerous small pores on the transverse bands in the *Cenocorixa bifida* play a role in the uptake of water as well as ions from the surrounding medium, and the labium is engaged in the active transport of ions from the medium into the haemolymph. 

In the present study the species of the Corixidae (except for the Cymatiinae), Micronectidae, and Diaprepocoridae have been observed to have the transverse bands with the semicircular grooves and pores and the set of sensory organs identical with that described by Lo and Acton [14] and Jarial [13]. At the same time, in the Cymatiinae the semicircular grooves with the pores have not been observed, and their set of sensory organs is slightly different. Among the ribbon-like sensilla the typical peg sensilla are present, and probably most of them can be an associated with osmotic and ionic regulation. 

It is reasonable to assume that most of the different types of the mechanosensilla occurring on the labium in other nepomorphans (Table 1(A)), although they are not equipped with pores, may also have the ability to permeate water as well as ions from the surrounding medium. 

It is surprising that many forms of various mechnosensilla of water bugs are presently being discovered while in some terrestial groups of bugs there seems to be less diversity with respect to the types of mechanosensilla (three to five types of chaetic sensilla) on the labium [15, 25, 43]. It is rather obvious that the numerous chetica sensilla play mainly a tactile function, whereas the remaining different types of mechanosensilla in the water bugs can also have the osmoregulatory function. 

With respect to their position, the trichoid setae (prioprereceptive sensilla) play a role in monitoring the position of the segments of the labium. They are commonly found in insects around joints, where they assist in prioprereception just as the campaniformia sensilla [4].

#### 4.4.2. Gustative and Contact Function on the Labial Tip

In nepomorphans, the main group of the sensilla at the tip of the labium is that of the uniporous papilla sensilla (PAS1, PAS2) (Table 1(C)). The presence of only two similar shapes of these sensilla on the labium is probably linked to the detection limit of the number of chemical stimuli through close and direct contact of the predator with its victim. 

Sensilla of a similar shape but more flattened have been found in the Gerromorpha (plate sensilla: triangular and oval ones) as well as dome-shaped sensilla, which represent the group of gustatory sensilla [Brożek and Zettel, in prep]. The presence of gustative peg sensilla has been observed in other studied heteropteran taxa of the Cimicomorpha (Reduviidae), and typical basiconic sensilla functioning as the gustative and contact-chemoreceptive sensilla have been observed in several species of the Pentatomomorpha. Moreover, the contact-chemoreceptive sensilla in many insects are represented by various forms of bristles, spots, cones, pits, and domes. Each of these has one external pore and is innervated by neurons with features characteristic of chemo- and mechanoreceptors [8]. Ultrastructural studies show that sometimes a mechanoreceptive dendrite may be associated with the sensilla that function as contact-chemoreceptive sensilla [7] (Table 1(B)). Such sensilla, with a terminal pore and a flexible socket and bent at the base of the stem, have been observed during our study; they should be regarded as mechano-chemoreceptive ones. Presently, it is certain that the trichoid sensilla have a terminal pore (Figure 4(a)) and are embedded in a flexible socket.

#### 4.4.3. The Labium and the Thermohygroreceptive Function

As for the peg-in-pit sensilla (PIP), their function has not been explicitly identified yet, however, drawing a conclusion from their presence on the labial tip (or another surface of the labium) it can be suggested that their function is possibly thermohygroreceptive (Table 1(D)). They are similar to the peg-no-pore sensilla [47] generally associated with thermohygr-perception, having a triad of neurons for cold, dry, and moist detection and described as occurring in many other insect orders [48–54]. Generally, these sensilla are present in low numbers and are distributed mainly on both lateral faces of the antennomeres, and only rarely on other parts of insect bodies. However, this type possibly occurs also on the labium. A confirmation of this has been found in terrestrial heteropterans and auchenorrhynchans, where the thermohygroreceptors of the peg-in-pit sensilla are spread over both the labial segments [3, 15, 25] and the antennae [6]. The geromorphan families seem to be morphologically similar to the nepomorphan ones regarding the labial structures mentioned above [Brożek and Zettel, in prep]. Moreover, Shoonhoven and Henstra [21] have provided further evidence that the twelve sensilla basiconica on the tip of the labium of *Dysdercus *(Pyrrhocoridae) are contact chemoreceptive and—in addition to their apparent role in sampling the substrate for food—they could also function as thermohygroreceptives because the bugs imbibe liquid food. In the water habitat, the thermohygroreceptive sensillum on the labium of the nepomorphans can be assumed to play some role also in feeding because this type of sensilla is more numerous on the antennae and used in order to control humidity and temperature during flights to other water bodies as well as during changes of temperature in a particular water body. 

### 4.5. Phylogenetic Implications and Conclusions

1. On the labium of water bugs there are more or less numerous sensilla of various shapes and sizes, classified as chemosensilla and mechanosensilla. According to the morphological characteristics of the labial sensilla it appears that they can provide the water bugs with information about tactile and gustative stimuli when they come in contact with a victim. Moreover, the types of these sensilla and their distribution on the labium also provide specific valuable systematic information regarding the subfamilies or families and allow insights into the complexity of character evolution.
2. The present comparative morphological study of the labial sensilla characters may enrich the scope of research focusing on the phylogeny of the Nepomorpha. Two latest hypotheses on infraorder families relationships that have been presented by Hebsgaard et al. [30] and Hua et al. [31] are in conflict regarding crucial points; that is, they are at odds with regard to recognition of the basal taxon in the phylogeny stem, as well as the position of the Pleidae. Nonetheless, a future comprehensive cladistic analysis using a range of morphological complexes and new characters of the labial sensilla will shed light on the evolution of the Nepomorpha. However, some preliminary phylogenetical remarks seem justified even at this point.
3. The greater part of the labium surface of water bugs is covered by “hair” layers. Nevertheless, several types of other mechanosensilla presently recognized in the taxa of the Nepomorpha might represent an evolutionary novelty. Presently, it is postulated that the cupola and peg sensilla are plesiomorphic features of the Belostomatidae, whereas the paddle-like sensillum in the Ranatrinae, the squamiform sensillum in the Nepinae, the clubbed-like sensillum in the Gelastocorinae, the chaetica sensillum with a bisected tip in the Nerthrinae, the star-like sensillum in the Aphelocheiridae, and finally the multilobe sensillum in some of the Naucoridae are an autapomorphy for each one of them, whereas the ribbon-like sensillum is synapomorphy for the Corixidae, Micronectidae, and Diaprepocoridae.
4. Substantial differences in the mechanosensilla set have been found between the subfamilies Gelastocorinae and Nerthrinae (Gelastocoridae). In the present study two autapomorphies (FRS, HLS) for the Gelastocorinae as well as two autapomorphies (CHB, COS) for the Nerthrinae have been evidenced. Will this difference have an influence on the phylogenetic value and rank of these taxa in the future?
5. Nevertheless, the short trichoid sensilla placed ventrally have been reported in only three taxa (Gelastocoridae, Ochteridae, and Aphelocheiridae), and this structure might represent an evolutionary novelty for them. There is evidence pointing to a close relationship between the Gelastocoridae and Ochteridae which, taken together, have formed the Ochteroidea, but the Aphelocheiridae are rather distal in relationship from those families and have formed the Aphelocheiroidea together with the Potamocoridae [30].
6. The presence of trichoid sensilla on the third segment is limited only to the subfamilies of the Naucoridae, and it is a satisfactory score (one synapomorphy) obtained to support the monophyly of the Naucoridae. The monophyly of the Naucoridae has been indicated in a previous study by Hebsgaard et al. [30].
7. Labial tip sensilla are assessed as a generally homogenous group of these structures in the Nepomorpha similar shapes and distribution of the sensilla (PAS1, PAS2, and PIP); only slight differences have been observed between the ground plan (Belostomatidae and Nepidae) and the remaining taxa.
8. The well visible contrast in the distribution of sensilla on the surface of the labium can be noticed between the Corixoidea and the remaining nepomorphan families. It seems that the shape of the labium strongly affects the distribution of the sensilla. In most nepomorphan taxa the sensilla are placed along the long axis of the labium, while in the Corixoidea (except for the Cymatiinae) these sensilla are placed in the transverse bands on the labium. Essentially, this fact is difficult to explain in the Cymatiinae. The triangular-shaped labium is an evolutionary novelty [2, 45] and is the same as in the remaining representatives of corixoids, but the sensilla are scattered unevenly (plesiomorphy). There exists a transverse pattern of distribution of the sensilla and an autapomorphy in the case of the Corixoidea (except for Cymatiinae). The Corixoidea have rather reached a new level of adaptation among nepomorphan taxa [2, 30, 32, 45, 55], and therefore they represent an advanced systematic position in contrast to the proposal of Hua et al. [31].

## Figures and Tables

**Figure 8:**
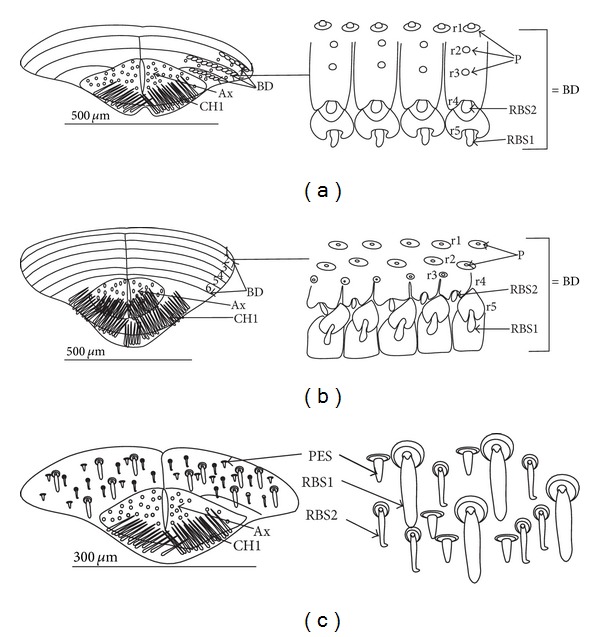
Types of distribution of the mechanosensilla on the dorsal surface of the labium in the Corixoidea. Sensilla placed in the transverse bands or scattered unevenly on the dorsal labial surface ((a) and (b)) and sensilla scattered unevenly on the labial surface (c). (a) Diaprepocoridae; *Diaprepocoris zealandiae*, two and half of bands, three rows (r1, r2, r3) of semicircular grooves with a pore, two rows of ribbon-like sensilla. (b) Corixidae; six bands, three rows (r1, r2, r3) of semicircular grooves with a pore, two rows of ribbon-like sensilla. (c) Cymatiinae; ribbon-like sensilla (RBS1, RBS2) and peg sensilla (PES).

**Table 1:** Terminology and classification of labial sensilla.

Category	Function	General characteristic sensilla of insects	Types of labial sensilla distinguished in the Nepomorpha (see results)
(A) *Mechanosensilla* Sensillum has 1 neuron. Dendrite is not branched and is attached by the cuticular sheath either to the center or to one side of the base of the hair. The cuticular sheath may continue beyond the end of the dendrite and insert into wall of the hair at various distances beyond the base [4, 8].	*Tactile* [6, 8, 24].	Mechanosensilla vary greatly in size and form and are shaped as thin hairs, chaetica, trichodea, trichobothria scale, filament, and peg. The shafts of sensilla protrude from the surface of cuticle, and their base is attached by an articulation membrane forming the flexible socket. The hair is usually drawn to a sharp tip and exteriorly may bear cuticular sculpturings such as grooves or spicules. Mechanosensilla have a wall-nonpore except the molting pore near the base [6, 8]. *NP*: nonpore.	Various shaped sensilla sunken into a *flexible socket*: chaetica sensilla (CH): (CH1 long, CH2 middle, and CH3 short), conical (COS)—prioprereceptor, basiconic (BAS), squamiforme sensillum (SQS), trichobothrium (TBS), clubbed-like (CBS), paddle-like (PDS), cupola-like (CUS), peg (PES), finger-like (FRS), freniale-like (HLS), chaetic sensillum with bisected tip (CHB), stars-like (STS), multilobes (MPS), ribbon-like (RBS), long (RBS1), and short (RBS2).

(B) *Contact-chemoreceptive sensilla (bimodal sensilla)* Sensillum has 3–10 neurons [28]. Chemosensitive dendrites are not branched and extend to tip of cone within dendrite sheath, which also ends in tip of cone. In addition, sensillum has a mechanosensitive neuron ending at the cuticle of the cone base and a tubular body [4].	*Gustatory and tactile* [6, 28].	The contact-chemoreceptors are short as well as hairs or various cones characterized by presence of a single pore at or close to the tip of the projection. These sensilla are placed on the surface and equipped with an articulation in a socket. They are often wider at base and gradually tapering towards the apex. The cuticular walls of these sensilla are smooth, with the molting pore near the base [4, 6]. *Tp*: with one terminal pore: uniporous.	Sensilla sunken in a *flexible socket*: trichoid sensilla (TRS): (long TRS1, short TRS2).

(C) *Chemoreceptive sensilla* *(unimodal sensilla)* Sensillum has 3–10 neurons [28]. Chemosensitive dendrites are the same as in contact-chemoreceptive sensilla but do not possess the mechnosensitive neuron [4].	*Gustatory* [6, 7, 15, 28].	The gustatory receptor can be located in a hair, papilla, basiconic, peg-, or plate-like elevations of the surface or beneath the flat areas that have a single terminal pore (TP-sensilla, uniporous). Generally, their base is sunken into inflexible sockets. The presence of the pore at the end of the sensillum shows their chemical function [6, 7]. *Tp*: with one apical pore: uniporous.	Sensilla sunken in an *inflexible socket*: papilla (PAS): (PAS1 with flattened tip, PAS2).

(D) *Chemoreceptive sensilla* Sensillum has 3 neurons. The dendrites of the two neurons extending into lumen of peg are hygrosensitive. A third dendrite ending below the base of the peg is thermosensitive. All dendrites are surrounded by dendrite sheath [4].	*Hygrosensitive and thermosensitive* [6].	Coeloconic sensilla are usually concealed in a pit of cuticle and it is without pores [6]. *NP*: nonpore.	Peg-in-pit sensilla (PIP): with an *inflexible socket* sunken in cavity.

**Table 2:** Detailed description of the number, shape, and distribution of the labial mechanosensilla and contact-chemoreceptive sensilla of the species representing nepomorphan taxa. Species marked with an asterisk (∗) are illustrated. Characters of all featured species have been analyzed and compared.

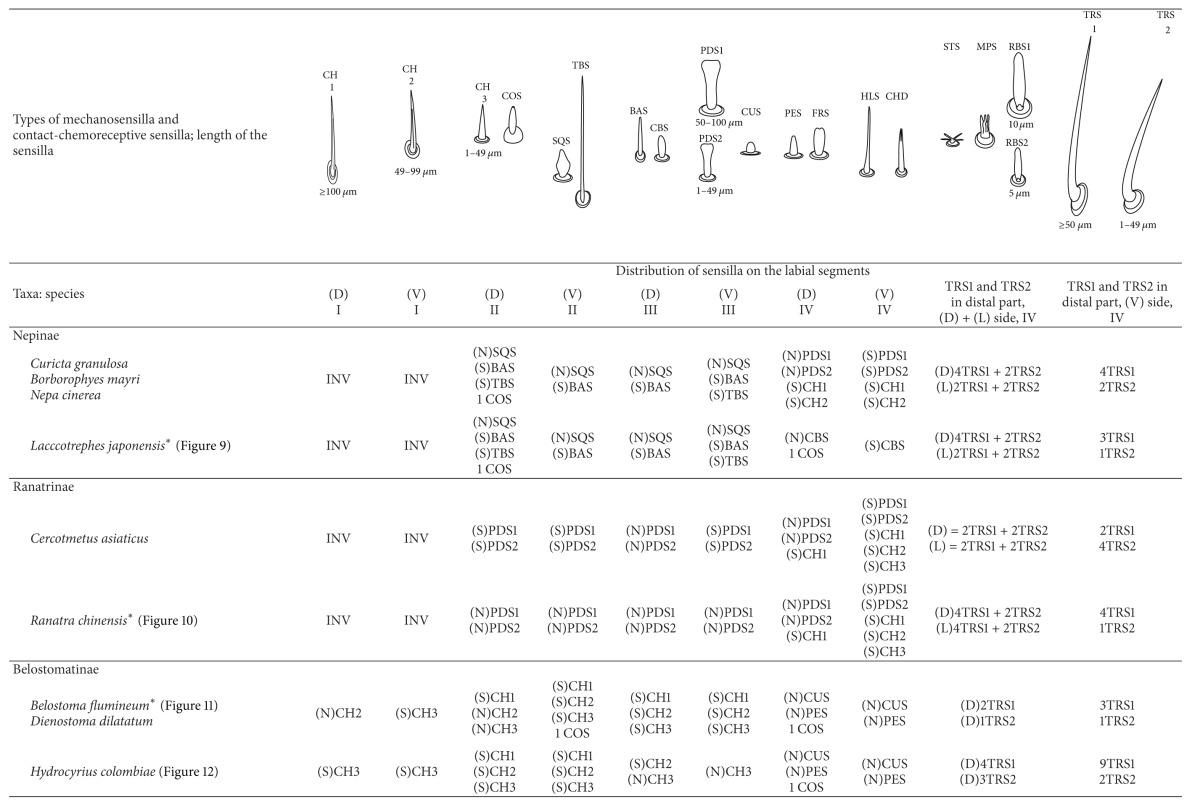 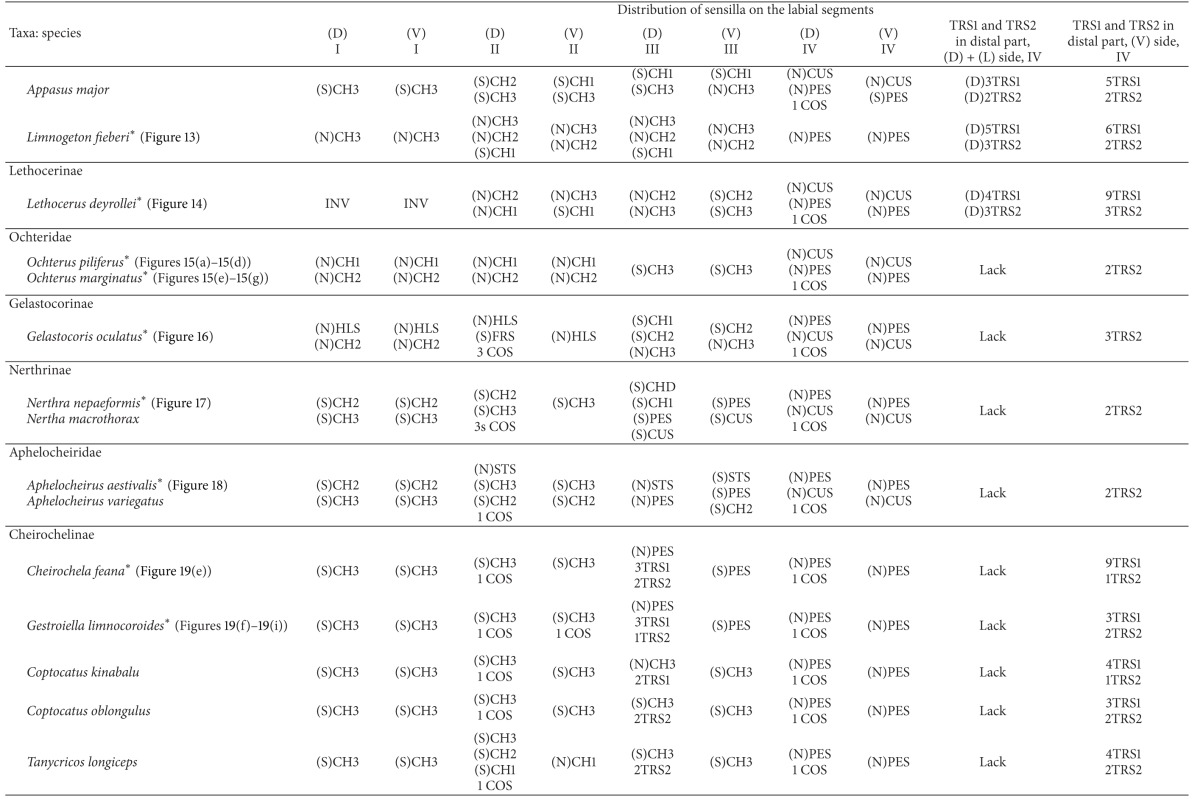 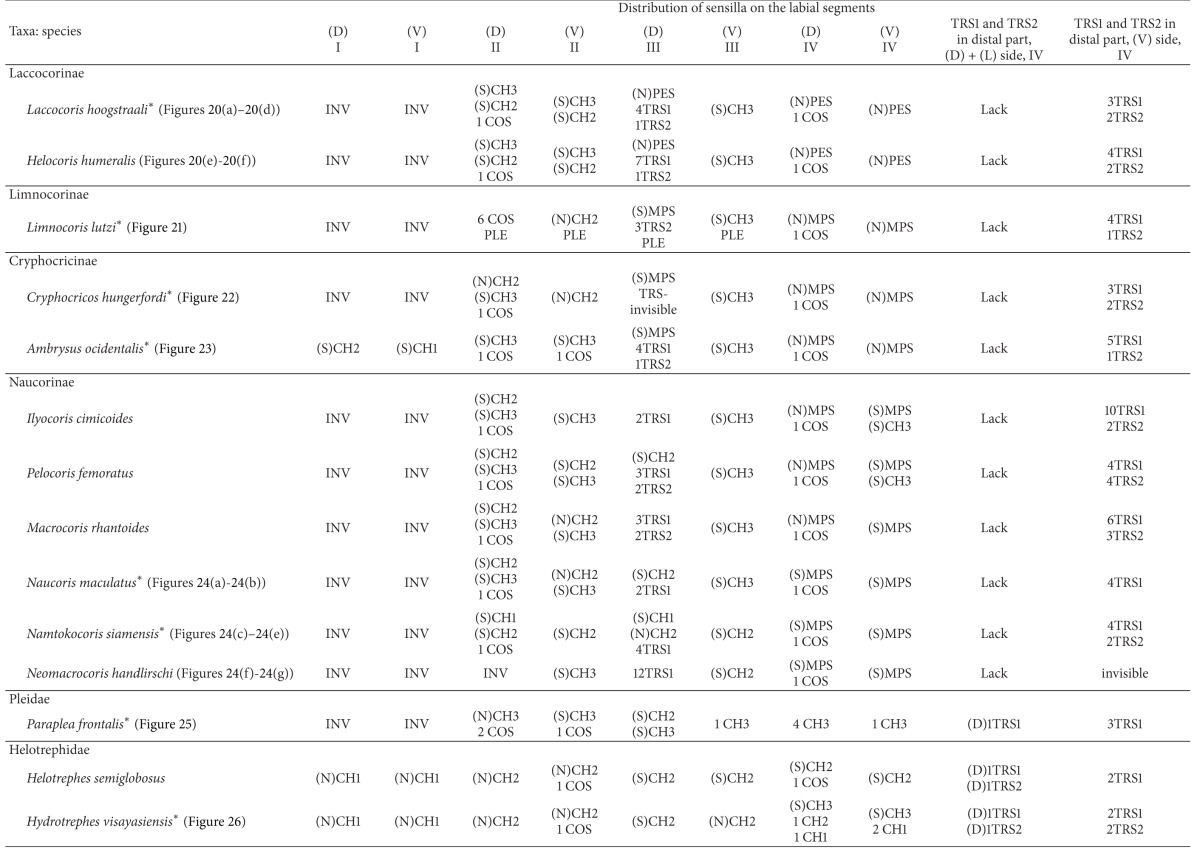 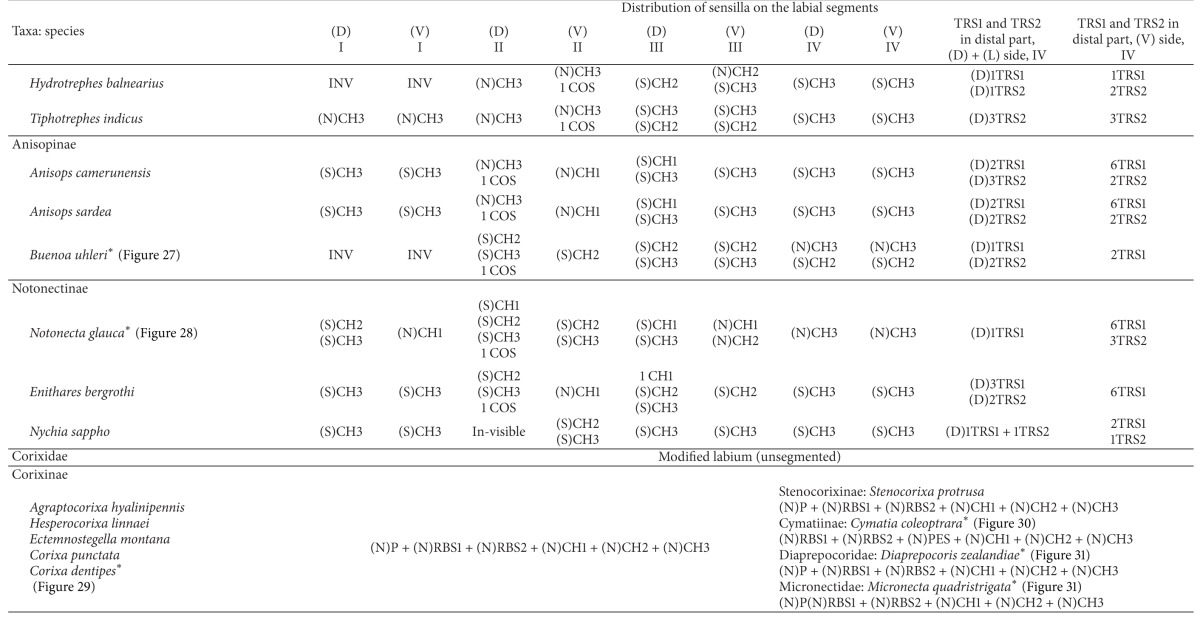

Symbol key: (D): dorsal side; (V): ventral side; (L): lateral side; (N): numerous sensilla; (S): several sensilla; INV: sensilla and I segment were invisible; 1–6 number of pairs COS and CH; I: first labial segment; II: second labial segment; III: third labial segment, IV: fourth labial segment. Abbreviation of names of the labial sensilla are explained in Table 1 and in the results.

## Appendix

Photographical documentation (SEM images) of the labial sensilla characters among the Nepomorpha subunits si shown in Figures 9–31.
